# Age- and gender-based social inequalities in palliative care for cancer patients: a systematic literature review

**DOI:** 10.3389/fpubh.2024.1421940

**Published:** 2024-09-04

**Authors:** Marina Rodríguez-Gómez, Guadalupe Pastor-Moreno, Isabel Ruiz-Pérez, Vicenta Escribà-Agüir, Vivian Benítez-Hidalgo

**Affiliations:** ^1^Hospital Clínico Universitario de Valencia, Valencia, Spain; ^2^Andalusian School of Public Health (EASP), Granada, Spain; ^3^CIBER of Epidemiology and Public Health (CIBERESP), Madrid, Spain; ^4^Instituto de Investigación Biosanitaria de Granada. Ibs. GRANADA, Granada, Spain; ^5^Fundación para el Fomento de la Investigación Sanitaria y Biomédica de la Comunidad Valenciana (FISABIO), Valencia, Spain

**Keywords:** palliative care, hospice and palliative care, gender, age, review – systematic

## Abstract

**Objectives:**

Cancer is a major public health problem worldwide, given its magnitude and growing burden, in addition to the repercussions on health and quality of life. Palliative care can play an important role improving quality of life and it is cost-effective, but some population groups may not benefit from it or benefit less based on age and gender inequalities. The aim of this systematic review was to analyze the available evidence on age- and gender-based social inequalities in access to and use of palliative care in cancer patients.

**Methods:**

A systematic review was conducted following the PRISMA guidelines. An exhaustive literature research was performed in Pubmed, CINHAL and Embase until November 2022 and were not restricted by language or date of publication. Eligible studies were observational studies analyzing the access and use of palliative care in cancer patients.

**Results:**

Fifty-three studies were included in the review. Forty-five analyzed age and 44 analyzed gender inequalities in relation to use of and access to palliative care. Our results show that older people receive poorer quality of care, worst symptom control and less preferences for palliative care. In relation to gender, women have a greater preference for the use of palliative care and generally have more access to basic and specialized palliative care services and palliative care facilities.

**Conclusion:**

This review reveals difficulties for older persons and men for access to key elements of palliative care and highlights the need to tackle access barriers for the most vulnerable population groups. Innovative collaborative services based around patient, family and wider community are needed to ensure optimal care.

## 1 Introduction

Cancer is one of the main causes of morbidity and mortality in the world. The International Agency for Research on Cancer estimates that in 2025 there will be 21.6 million new cases and 11.3 million deaths from cancer worldwide (1).

Advances in cancer diagnosis and treatment have led to an increase in survival and quality of life of cancer patients. But despite these advances, when cancer is diagnosed in advanced stages the chances of a cure are minimal and many people die of this disease. In such cases, palliative care plays an important role, as the aim of this type of care is to improve the quality of life of patients and their families by controlling pain and other symptoms and offering psychological, social and spiritual support (2). The origin of palliative care lies in the modern hospice movement, which is considered a philosophy of care for patients with advanced and terminal diseases that seeks to ensure a “good death” for those who are at the end-of-life (3).

Palliative care is currently seen in a broader perspective that includes the concept of early intervention and ongoing care suited to the needs of the sick and their families. According to the American Society of Clinical Oncology, palliative care should be initiated as early as possible from the time of advanced-stage cancer diagnosis, during treatment and after treatment. The objective is to provide quality care focused on improving symptom control, satisfying patients and their families, reducing use of healthcare services, such as visits to emergency departments, hospitalizations, admissions to intensive care units (ICUs), etc., and performance of inappropriate or unnecessary diagnostic and therapeutic procedures (4, 5).

However, there are difficulties inherent in any effort to develop quality indicators for palliative care, especially focused on end-of-life care. There is a limited evidence base and little consensus among experts and patients as to what constitutes optimal care, and the end-of-life period is hard to identify prospectively (4). There is scientific evidence of social inequalities in relation to cancer (6) and also in relation to palliative care, and the following have been observed: (a) disparities in the various approaches, such as variation in delivery of palliative radiotherapy to persons dying of cancer (7), intensity of end-of-life care (8) and variations in the use of hospice care (9), and (b) population groups that might not benefit from palliative care or would benefit to a lesser extent (10, 11).

These inequalities may be influenced by socio-demographic, clinical or geographical factors (12).

Among the socio-demographic variables, age and gender appear as essential factors in all chronic processes (13, 14).

Age discrimination may give rise to a limitation of healthcare opportunities for a large population group, given the aging of the population (15). It is clear that there are different complexities in care needs between some age groups and others (16, 17) and palliative care should be tailored to these needs to avoid this potential discrimination.

As for gender inequalities, the differences between men and women are not limited to differences in the presentation, identification and course of cancer; the social roles attributed to men and women also influence health and disease processes (18), including palliative care (19).

The objective of this study is to analyze the available evidence on age- and gender-based social inequalities in access to and use of palliative care in cancer patients.

## 2 Materials and methods

This study was part of a broader systematic review aimed to identify and analyze social inequalities in the access and use of palliative care in cancer patients. This review is part of a larger doctoral thesis^1^ and was not registered in any database prospectively. But to increase the transparency of the process, the review and its procedures were planned, conducted, and reported according to the Preferred Reporting Items for Systematic Reviews and Meta-Analyses (PRISMA) guideline (20), and sufficient details have been provided so that other researchers can reproduce the process.

### 2.1 Search strategy

A specific search strategy was developed for Pubmed using a combination of MeSH (Medical Subject Headings) terms and keywords from titles and abstracts, then the search was adapted for the other databases (CINAHL and Embase). The search strategy is shown in Supplementary Appendix 1. All the searches were performed up to November 2022 and were not restricted by language or date of publication. That is to say, there was no limit set on the number of years backward.

### 2.2 Study selection

Inclusion criteria: original studies with cross-sectional, cohort or case–control design targeting adult patients (≥18 years) with any type of cancer whose outcome variables measure access to or use of palliative care in the age and gender inequality axes.

### 2.3 Selection and data extraction process

The references obtained from the literature searches were loaded into the software Rayyan Qatar Computing Research Institute (Doha, Qatar), a free web application designed to facilitate the screening process for researchers working on systematic reviews, scoping reviews and other literature review projects (21). After deleting the duplicate references, we proceeded to screen the studies, first by reviewing the titles and abstracts and then by full-text review. The whole process was carried out by two reviewers (MRG and VEA) and doubts or discrepancies were discussed with a third reviewer (IRP).

Subsequently, forms were designed for data extraction from each study selected. The following information was recorded: title, author, year of publication, country of study, design, number of participants, target population, type of cancer and data collection instrument. The variables related to the inequality axes present and to the various approaches for assessing access to and use of palliative care were also recorded. Finally, the results found in the studies and the results of the risk-of-bias assessment of the studies were recorded.

### 2.4 Risk-of-bias assessment of the studies

An assessment of the methodological quality of each study was performed using the Newcastle-Ottawa scale (NOS) (22) adapted for case–control, cohort or cross-sectional studies.

The studies were classified into three groups according to the point score obtained in the analysis: high methodological quality (7–10 stars), moderate methodological quality (4–6 stars) and low methodological quality (<4 stars).

### 2.5 Data synthesis and analysis

According to a previous narrative review conducted by the authors (23), the terms and concepts used in the literature to refer to palliative care and the access and use to this type of care are very wide-ranging, since the same is true of the clinical or therapeutic measures to be adopted in patients. In this study, in order to standardize the terminology and facilitate analysis of the results, palliative care is grouped into five blocks, according to the approaches involved: (a) symptom management: pharmacological measures and/or cancer treatments for palliative purposes; (b) adequacy and quality of care: use of end-of-life healthcare services and aggressiveness end-of-life care (overuse of aggressive anticancer therapies or misuse of non-specific palliative care procedures or devices); (c) access to palliative care services: basic (supportive care) or specialized (end-of-life care [hospice care], palliative care programs, specialist palliative care units, etc.); (d) advance care planning: knowledge of palliative care, preferences and registration of these preferences (clinical history, prior instructions/advance directives) and (e) place of death: home, specialist palliative care centers, acute hospitals and nursing homes. This grouping was formulated on the basis of proposals made by other important authors and/or institutions (24–26).

The results were therefore synthesized and analyzed qualitatively, including a detailed description of the characteristics of the studies and of the inequality axes examined, a classification of the studies into the various approaches considered and an analysis of the inequalities, taking the findings of the studies into account.

## 3 Results

### 3.1 Study selection

**Figure 1:**
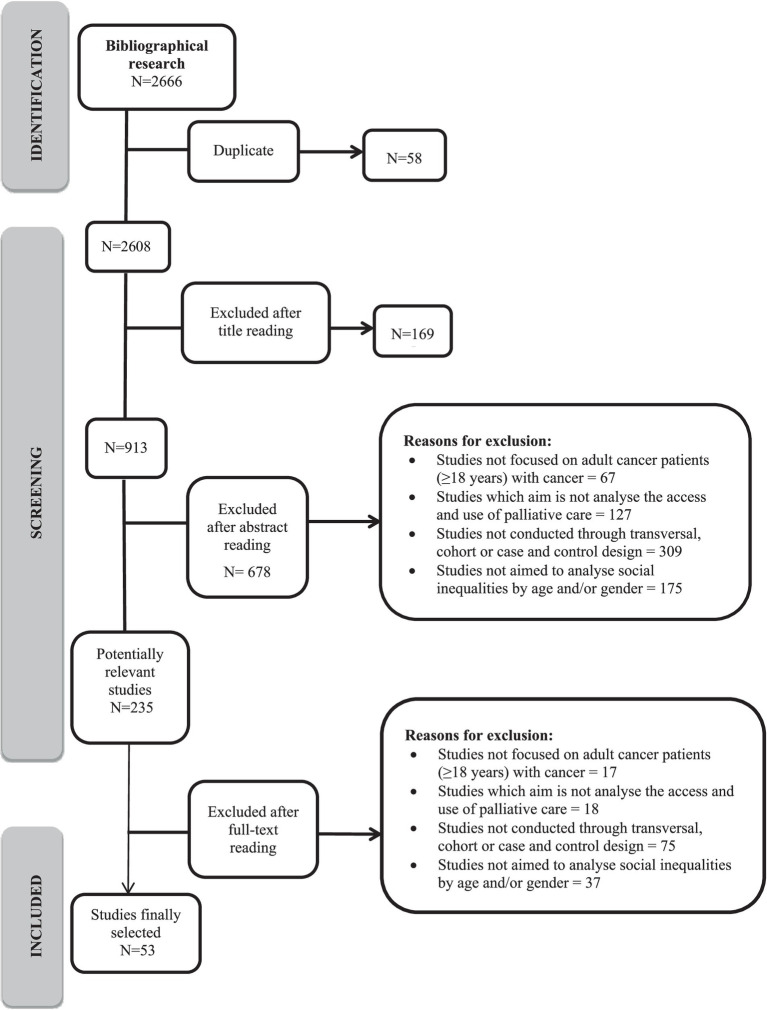
PRISMA flow diagram.

A total of 2,666 references were identified (Figure 1). Having excluded 58 duplicates, we proceeded to read the titles, abstracts and full texts. After reading full texts 182 studies were excluded. Finally, 53 studies that met the selection criteria and were suitable for the review were selected. Of these, 45 analyzed age (7, 8, 12, 14, 27–66) and 44 analyzed gender (7, 8, 12, 14, 27, 29–43, 45–68) in relation to use of and access to palliative care.

### 3.2 Study characteristics

The studies were published between 2001 and 2022. The years with the highest number of publications were 2020–2022 (20; 37.7%). The country in which the most studies were conducted was the United States (25), followed by Canada (7). Thirty-four studies analyze several types of cancer together, sometimes without identifying which. Of the remainder, 10 studies focus on gastrointestinal cancer, 2 on lung cancer, 2 on gynecological cancer, 2 on genitourinary cancer, 2 on central nervous system cancer and 1 on breast cancer.

Thirty-three of the studies follow a retrospective cohort design and the other 20 use a cross-sectional design (Table 1).

**Table 1:** Characteristics of the studies.

	*N* = 53	%
Year of publication		
2001–2003	6	11.3
2005–2008	4	7.5
2010–2014	8	15.1
2015–2019	15	28.3
2020–2022	20	37.7
Country of publication		
USA	25	47.2
Canada	7	13.2
Sweden	4	7.5
Australia	3	5.7
UK	3	5.7
Taiwan	2	3.8
China	2	3.8
Denmark	2	3.8
Germany	1	1.9
Italy	1	1.9
Norway	1	1.9
Puerto Rico	1	1.9
Spain	1	1.9
Type of cancer		
Various	34	64.1
Gastrointestinal	10	18.9
Lung	2	3.8
Gynecological	2	3.8
Genitourinary	2	3.8
Central nervous system	2	3.8
Mama	1	1.9
Study design		
Cohort	33	62.3
Cases and controls	-	-
Transversal	20	37.7
Instrument for collecting information		
Administrative data	42	79.2
Survey/questionnaire	3	5.7
Medical records	1	1.9
Various	7	13.2
Axis of inequality*		
Age	45	84.9
Gender	44	83
Methodological quality		
High	44	83.1
Moderate	9	16.9
Low	–	–
Palliative care approaches*		
Symptom management	9	17.0
Adequacy and quality of care	10	18.9
Palliative care services	26	49.1
Advance care planning	5	9.4
Place of death	17	32.1

*The total does not necessarily add up to 53, as the classification system is based on non-exclusive categories.

### 3.3 Methodological quality

A total of 83.1% of the studies were assessed as being of high methodological quality with low risk of bias and 16.9% of moderate methodological quality (9, 14, 39, 47, 53, 57, 58, 62, 64) (Table 2).

**Table 2:** Methodological quality evaluation.

**Authors**	**Selection**	**Comparability**	**Outcome/Exposition**	**Total**
Bergqvist et al. (2022)	*****	**	***	10
Åsli et al. (2018)	****	**	***	9
Barbera et al. (2010)	****	**	***	9
Burge et al. (2005)	****	**	***	9
Colibaseanu et al. (2018)	****	**	***	9
Dumbrava et al. (2018)	****	**	***	9
Hegagi et al. (2022)	****	**	***	9
Huang et al. (2001)	****	**	***	9
Johnston et al. (2001)	****	**	***	9
Lai et al. (2020)	****	**	***	9
Lindskog et al. (2022)	****	**	***	9
Neergaard et al. (2012)	****	**	***	9
Öhlén et al. (2017)	****	**	***	9
Sedhom et al. (2021)	****	**	***	9
Alterio et al. (2021)	****	**	**	8
Burge et al. (2008)	***	**	***	8
D’Angelo et al. (2020)	****	**	**	8
Deeb et al. (2021)	***	**	***	8
Han et al. (2021)	***	**	***	8
Heller et al. (2019)	****	**	**	8
Lavergne et al. (2011)	****	**	**	8
Lee et al. (2021)	****	**	**	8
Li et al. (2020)	***	**	***	8
Perry et al. (2021)	****	**	**	8
Sharp et al. (2018)	*****	**	*	8
Tang et al. (2012)	****	**	**	8
Wong et al. (2014)	****	**	**	8
Adsersen et al. (2021)	****	**	*	7
Craigs et al. (2018)	****		***	7
Davaro et al. (2021)	****	**	*	7
Hu et al. (2021)	****	**	*	7
Hunt et al. (2001)	***	**	**	7
Hunt et al. (2002)	***	**	**	7
Jackson et al. (2022)	****	**	*	7
Jin et al. (2022)	****	**	*	7
Koroukian et al. (2017)	****	**	*	7
López-Valcarcel et al. (2019)	*****		**	7
Maddison et al. (2012)	****	**	*	7
Milki et al. (2021)	****	**	*	7
Okafor et al. (2017)	***	**	**	7
Ramos-Fernánez et al. (2022)	****	**	*	7
Rosenfeld et al. (2018)	****	**	*	7
Rubens et al. (2019)	****	**	*	7
Watanabe-Galloway et al. (2014)	***	**	**	7
O’Mahony et al. (2021)	****	**	*	6
Papke et al. (2007)	***		***	6
Saeed et al. (2018)	***	**	*	6
Saeed et al. (2019)	***	**	*	6
Sharma et al. (2015)	***	*	**	6
Gatrell et al. (2003)	***		**	5
Lackan et al.	****		*	5
Nayar et al. (2014)	***		**	5
Koffman et al. (2007)	**	*	*	4

### 3.4 Synthesis of results

The Tables 3–5 shows the results of the classification of the studies finally included according to the type of palliative care approach and the result variables examined and grouped by common characteristics, and in Supplementary Appendix 2 these can be seen in more detail.

**Table 3:** Number of studies identified by inequality access and type of approach.

Type of palliative care approach	Inequality axis	
	Gender	Age
Symptom management	5	9
Adequacy and quality of care	9	10
Access to PC services (basic or specialized)	20	23
Advance care planning	5	2
Place of death	15	15

**Table 4:** Approaches to analyze the access and use of palliative care of included studies.

PC approaches*	Outcome measure	*N* = 53	%
**Symptom management**		**9**	**17.0**
	Consultation and/or treatment with palliative intent	9	17.0
Adequacy and quality of care		**10**	**18.9**
	Use of health services at EOL (aggressiveness of care at EOL)	10	18.9
**Palliative care services**		**26**	**49.1**
	Basic care	4	7.5
	Specialized care	25	47.2
	Specialized PC units	4	7.5
	Inpatient care	11	20.7
	EOL care (hospice)	7	13.2
	PC programs	2	3.8
	Ambulatory	1	1.9
**Advance care planning**		**5**	**9.4**
	PC knowledge	1	1.9
	PC preferences	3	5.7
	Advance directives	1	1.9
**Place of death**		**17**	**32.1**
	Acute care (hospital)	7	13.2
	Home	1	1.9
	Various places	9	17

* The total does not necessarily add up to 53, as the classification system is based on non-exclusive categories. PC, palliative care; EOL, end of life.

**Table 5:** Study classification by PC approaches.

PC approaches*	Outcome measure	Authors
Symptom management (*N* = 9)	Consultation and/or treatment with palliative intent (*N* = 9) (radiotherapy, chemotherapy and/ or surgery)	Alterio et al. (28), Åsli et al. (29), Colibaseanu et al. (34), Davaro et al. (69), Dumbrava et al. (38), Huang et al. (75), Johnston et al. (7), Lavergne et al. (12), Wong et al. (71)
Adequacy and quality of care (*N* = 10)	Use of health services at EOL (*N* = 10) -Aggressiveness of care at EOL (emergency room visits, intensive care unit admission, hospital admission, etc.)	Bergqvist et al. (31), Deeb et al. (37), Koroukian et al. (8), Lindskog et al. (50), Maddison et al. (52), Nayar et al. (53), Perry et al. (59), Ramos-Fernández et al. (60), Tang et al. (72), Watanabe-Galloway et al. (66)
Palliative care services (*N* = 26)	Basic care (*N* = 4)	
	Support care (*N* = 4) (Home care, social support, telephone monitoring, primary care, support groups…)	Barbera et al. (30), Craigs et al. (35), Jin et al. (46), Sharp et al. (65)
	Specialized care (*N* = 25)	
	Specialized PC units (*N* = 4)	Adsersen et al. (27), Bergqvist et al. (31), Hegagi et al. (41), Lindskog et al. (50)
	Inpatient care (*N* = 11) (during hospital admission)	Craigs et al. (35), Han et al. (40), Heller et al. (42), Jackson et al. (45), Jin et al. (46), Lee et al. (49), Milki et al. (73), Okafor et al. (56), Rosenfeld et al. (74), Rubens et al. (61), Sharma et al. (64)
	EOL care (*N* = 7) (hospice care)	Hunt et al. (67), Koroukian et al. (8), Lackan et al. (9), Lai et al. (48), Nayar et al. (53), Tang et al. (72), Watanabe-Galloway et al. (66)
	PC programs (*N* = 2)	Burge et al. (33), Maddison et al. (52)
	Ambulatory (*N* = 1)	Jin et al. (46)
Advance care planning (*N* = 5)	PC knowledge (*N* = 1)	Koffman et al. (47)
	PC preferences (*N* = 3)	Hu et al. (43), O’Mahony et al. (57), Saeed et al. (14)
	Advance directives (*N* = 1)	Saeed et al. (62)
Place of death (*N* = 17)	Acute care (*N* = 7) (hospital)	Barbera et al. (30), Bergqvist et al. (31), Koroukian et al. (8), Li et al. (68), Lindskog et al. (50), Maddison et al. (52), Tang et al. (72)
	Home (*N* = 1)	Neergaard et al. (54)
	Various places (*N* = 9) (acute hospital, home, socio-health centers or PC centers)	Burge et al. (32), D’Angelo et al. (36), Gatrell et al. (39), Hegagi et al. (41), Hunt et al. (44), López-Valcarcel et al. (51), Öhlén et al. (55), Papke et al. (58), Sedhom et al. (63)

*The total does not necessarily add up to 53, as the classification system is based on non-exclusive categories. PC, palliative care; EOL, end of life.

Forty-four studies analyze how gender influences access to and use of palliative care. Five analyze access to symptom management (7, 12, 29, 34, 38), 9 adequacy and quality of care (8, 31, 37, 50, 52, 53, 59, 60, 66), 20 access to palliative care services (8, 27, 30, 33, 35, 40–42, 45, 46, 48–50, 52, 53, 56, 61, 64–66), 5 advance care planning (14, 43, 47, 57, 62) and 15 the place of death (8, 30–32, 36, 39, 44, 50–52, 54, 55, 58, 63, 68).

Of the 45 studies identified that examine the effect of age on access to and use of palliative care, 9 analyze access to care for symptom management (7, 12, 28, 29, 34, 38, 69–71), 10 adequacy and quality of care (8, 31, 37, 50, 52, 53, 59, 60, 66, 72), 23 access to palliative care services (8, 9, 27, 30–32, 35, 40–42, 45, 48–50, 52, 53, 61, 65–67, 72–74), 2 advance care planning (14, 62) and 15 the place of death (8, 30–32, 36, 39, 41, 50, 52, 54, 55, 58, 63, 67, 72).

### 3.5 Age and symptom management

Of the 9 studies that assess the effect of age on access to care for symptom management (access to consultation and/or cancer treatment for palliative purposes: radiotherapy, chemotherapy, palliative surgery and pain management), 7 show that access to such care is lower in older patients, especially those over the age of 80, compared to younger patients (7, 12, 29, 34, 38, 71, 75). Alterio et al. (28) and Davaro et al. (69) find no age differences in reception of palliative care in patients with metastasis and advanced cancer.

### 3.6 Age and adequacy and quality of care

Of the 10 studies that assess the aggressiveness of end-of-life care according to age, and therefore the adequacy and quality of care, 8 report fewer emergency department visits, hospital admissions, curative treatment with chemotherapy, etc., and therefore lesser therapeutic aggressiveness in older patients, especially those aged over 80–85 years (8, 31, 37, 53, 59, 60, 66, 72). Three studies find no differences in respect of age and emergency department visits (50, 52, 73).

Lindskog et al. find no differences in hospital admissions, but their study is restricted to the last 3 months of life (50), and Perry et al. find no differences in ICU admissions, but in relation to the last month of life (59).

### 3.7 Age and access to palliative care services

Twenty-three studies analyze the effect of age on access to palliative care services.

In 12 studies older patients were less likely to have access to basic palliative care (supportive care, home care, telephone follow-up, etc.) (30, 35, 65) and to specialized palliative care (specialist palliative care units, palliative care programs and hospice care) (9, 27, 31, 33, 35, 48, 50, 52, 66).

In contrast, 9 studies show more frequent use of palliative care in older patients (40–42, 49, 53, 61, 72–74) and 4 find no statistically significant differences in access to basic palliative care (35, 65) and to specialized palliative care (hospice care and palliative care during hospitalization [inpatient palliative care]) (8, 45).

### 3.8 Age and advance care planning

Two studies examine how age influences opinions on advance care planning. Saeed et al. conclude that completion of advance directives on care is preferred by older patients compared to younger patients (62) and that patients over 65 show less preference for palliative care compared to younger patients (14).

### 3.9 Age and place of death

Fifteen studies analyze the association between place of death and age and 8 show that older patients die at home less often (30, 31, 39, 44, 55, 58, 63, 72).

With regard to dying in hospital, older patients did so more often in 2 studies (39, 44) and less often in 5 (30, 31, 55, 63, 72).

Six studies find no statistically significant relationship between age and dying in acute hospital or dying out of hospital (8, 32, 41, 50, 55).

### 3.10 Gender and symptom management

Five studies evaluate the association between gender and access to care for symptom management (access to consultation and/or cancer treatment for palliative purposes [radiotherapy]) (7, 12, 29, 34, 38). Two of these studies show that women are less likely to receive palliative radiotherapy compared to men (12, 29). The other three studies (7, 34, 38) find no statistically significant differences in access to consultation and/or radiotherapy, chemotherapy and surgery.

### 3.11 Gender and adequacy and quality of care

Nine studies assess suitability of care according to gender, analyzing aggressiveness of end-of-life care (hospital admissions, ICU admissions, visits to emergency departments, systemic therapy: chemotherapy, mechanical ventilation, etc.).

Four studies show that women have better therapeutic suitability because they receive less aggressive care (50, 53, 60, 66). As for admissions, Bergqvist et al. show a higher number of hospitalizations in women with breast cancer than in men with prostate cancer (31).

Seven studies find no differences in chemotherapy treatment (8, 59), emergency department visits (8, 37, 50, 52, 53, 59), hospital admissions (59, 66), ICU admission (59) or use of invasive treatments (37, 59).

### 3.12 Gender and access to palliative care services

Twenty studies consider access to palliative care services according to gender. Ten of them show that being a woman increased the chances of having access to palliative care, both basic (30, 46) and specialized (27, 33, 35, 48, 49, 53, 56, 66), compared to men.

Three studies (30, 35, 46) show greater access for women to palliative care plans such as home visits, inpatient palliative care and social support. In the study by Sharp et al. (65) it was men that were more likely to have access to personalized care plans.

Thirteen studies find no gender differences in access to basic (30, 35, 46, 65) or specialized (40, 42, 45, 46, 61, 64) palliative care, specialist palliative care units (41, 50), outpatient palliative care (46), hospice care (8) and registration in a palliative care program (52).

### 3.13 Gender and advance care planning

Five studies analyze how gender influences preferences on care planning (14, 43, 47, 57, 62).

The studies by Saeed et al. (14), O’Mahony et al. (57) and Hu et al. (43) show that women have a greater preference for palliative care and less for the use of life-prolonging invasive treatments (mechanical ventilation) compared to men, but in some cases women prefer decisions on care to be initiated by others (healthcare professionals). Two studies find no statistically significant differences in relation to knowledge of palliative care (47) and preferences regarding care objectives or cardiopulmonary resuscitation orders (57).

### 3.14 Gender and place of death

Fifteen studies show results on gender and place of death.

Four show that women die less often in acute hospitals (30, 39, 51, 63). They are more likely than men to die out of hospital (32), at home (51), in a specialist palliative care center (44, 51, 63) and in nursing homes (39, 44, 58, 63).

Hunt et al. show that women, compared to men, are more likely to die in a private hospital than in a public hospital (44).

Eight studies found no relationship between gender and dying at home (36, 44, 54), in specialist palliative care centers (36), in acute hospitals (8, 50, 52, 55, 68), in nursing homes (55) or out of hospital (41).

## 4 Discussion

Although palliative care services have increased, it is equally important to understand whether these services are used by patients who really need them. The social determinants of health influence access to and use of palliative care (52, 76, 77), but no reviews have been published on this that allow us to assess whether or not inequalities exist. This is the first systematic literature review to analyze palliative care in respect of two axes of inequality (age, gender) and of five specific ways of addressing access to and use of this type of care in adult cancer patients. The main results to be highlighted are the following: older people receive worse care for symptom control and poorer quality of care and show less preference for palliative care; there are no conclusive results on place of death. With regard to gender, women have a greater preference for the use of palliative care, generally have more access to basic and specialized palliative care and die less often in hospitals and more in palliative care facilities.

The contradictory results found may be explained by the fact that the use of palliative care cannot be addressed without taking account of comorbidities and the presence or absence of metastasis (42, 61) and even the type of cancer. Moreover, survival time in a specialized palliative care setting is a good indicator of timely referral to the service and is often associated with less aggressive treatments (use of chemotherapy, emergency department visit, intensive care admission) (36).

On the other hand, there is literature on the need to use quality indicators to improve comparability in studies, among other things (4), but in spite of this, a large degree of variability has been observed in the time windows considered. For example, Colibaseanu et al. consider patients who survived for less than 6 months (34), Dumbrava et al. restrict their analysis to patients who did not die within 30 days of diagnosis (38), Lavergne et al. study palliative radiotherapy (PRT) treatment received in the last 9 months of life (12), Wong et al. examine the reception of chemotherapy within 14 days of death (71) and Asli et al. consider patients who had PRT at least once during the last two years before death (29). Bergqvist et al. use data on palliative care services received for the 3 months preceding the date of death (31) and Tang et al. examine underuse of hospice services as measured by lack of or very late referral to hospice (3 days before death) (72).

Most of the studies use administrative databases, and as with many studies relying on administrative sources of healthcare data, some of the services identified in the administrative data have not been validated with chart review data or individualized information.

We find that 10 studies independently looked at gastrointestinal cancers. The type of cancer studied may be determined by its magnitude and burden. Gastrointestinal cancers represent a large proportion of new cases and deaths from cancer worldwide, for example, colorectal cancer is one of the most incident and deadly cancer (78). Also, the aggressiveness and the stage of cancer can determine the study of a type of cancer, for example pancreatic cancer is usually diagnosed in advanced stages with poor prognoses and high palliative needs. Therefore, there is a greater need for evaluating the state of palliative care in these types of cancer. In addition, gastrointestinal cancer encompasses different types of cancer related to the diverse organs that are part of the gastrointestinal system.

Most of the studies were carried out in the USA. It is possible that the American health system (unlike universal coverage) could create more inequalities than other health systems, thus generating more interest among the country’s scientific community. It is also possible that the development level of low-income countries corresponds to the underdevelopment of their health systems or financial difficulties. Generally, there is limited access to palliative care due to distances, poor accessibility to healthcare in general, and cultural beliefs and attitudes toward illness (79). Peeler’s review highlights that the northern and central regions of Africa are underrepresented in the published scientific literature. They found that almost all African countries lacking published literature on primary palliative care also had no known palliative care activities or were in very early stages of capacity development (80).In this review we have seen that many studies continue to analyze the suitability of care and end-of life palliative care and that few studies have focused on studying advance planning of care, another important issue within palliative care, despite the change in the palliative care model that suggests early integration of palliative care (81).

Another essential issue within palliative care is the place of death, and especially dying at home (82).

However, this is not always possible or desirable and depends on the availability of resources (83). The best choice is one that is mutually agreed and takes account of the preferences and wishes of the patient, family members and caregivers (84) and the best place to die is the one that matches those preferences (85). In the studies reviewed here preferences are not taken into account or not mentioned and only the place where the patients die is analyzed. Moreover, the outcome measure is the actual place of death, which is not necessarily where patients spend most of their last months. Death in the hospital does not rule out the possibility that they were dying somewhere else up until their last days.

Very few of the studies reviewed were designed to examine differences between men and women and the gender variable was included as an adjustment variable. The importance of gender has recently been highlighted as a source of inequalities in palliative care (11, 86, 87).

The social roles ascribed to men and women in a given society influence attitudes and beliefs about health and disease and may guide healthcare decisions (88). It must be borne in mind that there are differences between men and women in the presentation, detection and evaluation of the disease and the related symptoms (86, 88) and that women, for cultural reasons, may prefer decisions about their health to be initiated by others (43), which could also determine access to palliative care and might explain the lower access of women to care for symptom control as well as cancer treatment for palliative purposes (14).

The limitations of this review arise from the variability of the studies analyzed, which makes it difficult, in many cases, to obtain conclusive results. On the other hand, however, it has allowed us to point out the difficulties of the study proposed and the need to explore the disparities identified here in more depth. Taking only two axes of inequality into account, our study reveals difficulties faced by structurally vulnerable population groups in gaining access to key elements of palliative care and highlight the need to tackle barriers to access.

Future interventions should promote that all cancer patients receive high-quality palliative care, regardless of their age or gender, respecting their individual needs and promoting equity in healthcare. It is necessary to ensure that professionals understand the particularities of palliative care in older patients, including polypharmacy, frailty, and comorbidities. Support services are also required to address the specific concerns of each gender, such as the caregiver role traditionally associated with women or the stigma of vulnerability in men.

This review reveals difficulties for older persons and men for access to key elements of palliative care and highlights the need to address access barriers for the most vulnerable population groups. The inequalities identified in this review are not just the responsibility of the hospice movement. Innovative collaborative services based around patient, family and wider community are needed to ensure optimal care for all. For some groups, therefore, lower use of hospice services may reflect care preferences and choices rather than inequality of provision.

## Footnotes

^1^ https://idus.us.es/handle/11441/148587
