# Supplementary material for: Age- and gender-based social inequalities in palliative care for cancer patients: a systematic literature review
Source: Front Public Health. 2024 Sep 4;12:1421940. doi: 10.3389/fpubh.2024.1421940 (PMC11408182; doi:10.3389/fpubh.2024.1421940)
Supplement: Supplementary file 1 [file Data_Sheet_1.docx]

**APPENDIX 1. Register of bibliographic search**

**Terms**

1)“Sexual minorit*” OR “Gender Minorit*” OR Homosexuality OR Bisexual* OR LGBT OR GLBT OR LGB OR GLBTQ OR “gender role*” OR "Gender identity" OR "Healthcare Disparities" OR “Healthcare Care Disparit*” OR “Healthcare Inequalit*” OR “Health Status Disparit*” OR Poverty OR “Vulnerable Population*” OR "Medical Indigency” OR Rural OR “low-income” OR inequit* OR inequalit* OR disadvantage* OR disparit* OR “inner-city” OR “Social Condition*” OR “Social Class*” OR “Ethnic Group*” OR "Cultural Diversity" OR "Minority Health" OR “Minority Group*” OR "Socioeconomic factors" AND

2) Neoplasms OR CANCER OR Oncology AND

3)"Terminal Care" OR "Palliative Care"

***PUBMED**

**Mesh** terms:

- Cancer[TW] including Cancer pain Mesh
- Oncology[TW] including Oncology nursing, Psicooncology Mesh

**Search Strategy:**

((("Neoplasms"[Mesh] OR CANCER[TW] OR Oncology[TW]) AND

("Terminal Care"[Mesh:noexp] OR "Palliative Care"[TW] OR "terminal care"[title/abstract]) AND

(Sexual minorit*[Title/Abstract] OR Gender Minorit*[Title/Abstract] OR "sexual and gender minorities"[Mesh] OR Homosexuality[TW] OR Bisexual*[Title/Abstract] OR LGBT[Title/Abstract] OR GLBT[Title/Abstract] OR LGB[Title/Abstract] OR GLBTQ[Title/Abstract] OR gender role*[Title/Abstract] OR "Gender identity"[Mesh] OR "Healthcare Disparities"[TW] OR Healthcare Care Disparit*[Title/Abstract] OR Healthcare Inequalit*[Title/Abstract] OR Health Status Disparit*[TW] OR "Poverty Areas"[TW] OR Poverty[TW] OR Vulnerable Population*[Title/Abstract] OR "Medical Indigency"[Title/Abstract] OR Rural[TW] OR low-income[Title/Abstract] OR inequit*[Title/Abstract] OR inequalit*[Title/Abstract] OR disadvantage*[Title/Abstract] OR disparit*[Title/Abstract] OR inner-city[Title/Abstract] OR Social Condition*[Title/Abstract] OR Social Class*[Title/Abstract] OR Ethnic Group*[Title/Abstract] OR "Cultural Diversity"[Title/Abstract] OR "Minority Health"[MH] OR Minority Group*[TW] OR "Socioeconomic factors"[TW])) AND Humans[Mesh]) Sort by: Best Match Filters: Human
