# Supplementary material for: Age- and gender-based social inequalities in palliative care for cancer patients: a systematic literature review
Source: Front Public Health. 2024 Sep 4;12:1421940. doi: 10.3389/fpubh.2024.1421940 (PMC11408182; doi:10.3389/fpubh.2024.1421940)
Supplement: Supplementary file 2 [file Data_Sheet_2.docx]

APPENDIX 2. Characteristics of the studies analysing social inequalities bye AGE and palliative care approaches

| **Author/ Year/Country** | **Study design/ N** | **Study population** | **Collection method (extraction of information)** | | **Adjustment variables** | **Outcome measure** | **Main results** | **NOS^§^** |
| --- | --- | --- | --- | --- | --- | --- | --- | --- |
| **Symptom management** | | | | | | | | |
| **Alterio et al.**  **2021**  **USA** | Retrospective cohort/ 27263 | Patients ≥ 18 years diagnosed with hepatocellular carcinoma stage IV between 2004-2016 | | National cancer registry | -Race  -Comorbidities  -Health insurance  -Income  -Education level  -Year of diagnosis  -Treatment facility | Receipt of surgery, radiotherapy, chemotherapy with palliative intent and/or pain management | **OR 1.49 (1.09-2.04)** patients 45-59 years vs. patients <45 years.  OR 1.37 (1.00-1.87) patients 60-74 years vs. patients <45 years.  OR 1.28 (0.93-1.76) patients >75 years vs. patients <45 years. | **H** |
| **Åsli et al. 2018**  **Norway** | Retrospective cohort/ 25287 | Patients who died with cancer (excluding non-melanoma skin cancer) between 1 July 2009 -31 December 2011 in Norway | | National registry | -Gender  -Cancer site  -Survival  -County of residence  -Previous curative radiotherapy  -Education level  -Household income  -Radiotherapy facility at diagnosing hospital  -Travel distance | Receipt of palliative radiotherapy in the last two years of life | OR 0.86 (0.72-1.02) patients 50-59 years vs. patients <50 years.  **OR 0.81 (0.69-0.95)** patients 60-69 years vs. patients <50 years.  **OR 0.57** **(0.48-0.67)** patients 70-79 years vs. patients <50 years.  **OR 0.31** **(0.27-0.37)** patients >80 years vs. patients <50 years. | **H** |
| **Colibaseanu et al. 2018**  **USA** | Retrospective cohort/ 287923 | Patients who died of colorectal cancer between 2004 and 2013 | | National cancer registry | -Year of diagnosis  -Gender  -Ethnicity/race  -Health insurance  -Income  -Education level  -Urban/rural residence  -Travel distances  -Comorbidities  -Hospital characteristics  -Hospital region  -Percentage of patients receiving palliative care  -Tumor characteristics | Receipt of surgery, radiotherapy, chemotherapy with palliative intent and/or pain management | ***Survival <6 months***  **OR 0.94 (0.91-0.97)** older patients vs. younger patients.  ***Survival 6-24 months***  **OR 0.94** **(0.91-0.98)** older patients vs. younger patients.  ***Survival >24 months***  OR 0.98 (0.92-1.05) older patients vs. younger patients. | **H** |
| **Davaro et al. 2021**  **USA** | Retrospective cohort/  Locally advanced cancer: 385  Metastatic: 279 | Patients diagnosed with advanced penile cancer between 2004-2015 | | National cancer registry | -Race  -Comorbidity  -Treatment facility  -Health insurance  -Income  -Year of diagnosis | Receipt of surgery, radiotherapy, chemotherapy with palliative intent and/or pain management | **Patients with metastasis**  **OR 0.971 (0.946-0.997)** older patients vs. younger patients.  **Patients with locally advanced cancer**  0.998 (0.964-1.034) older patients vs. younger patients. | **H** |
| **Dumbrava et al. 2018**  **Australia** | Retrospective cohort/ 1173 | Patients diagnosed with unresectable pancreatic adenocarcinoma between July 2009 and June  2011, residents in Queensland and New South Wales, Australia | | Medical records and National cancer registry | -Gender  -Charlson comorbidity index  -ECOG scale | NOT receipt palliative chemotherapy during the first year following diagnosis | OR 1.10 (0.75-1.61) patients 60-69 years vs. patients <60 years.  **OR 2.05 (1.40-3.00)** patients 70-79 years vs. patients <60 years.  **OR 8.21** **(5.33-12.64)** patients >80 years vs. patients <60 years. | **H** |
| **Huang et al.**  **2001**  **Canada** | Retrospective cohort/ 193253 | Patients > 20 years who died of cancer between 1986 and 1995 | | Regional cancer registry and cancer centres | -Year of death  -Income  -Hospital at diagnosis  -County  -Region | Receipt palliative radiotherapy in the last two years of life | **OR 2.01** **(1.93-2.10)** patients 70-79 years vs. patients >80 years.  **OR 3.04** **(2.92-3.17)** patients 60-69 years vs. patients >80 years.  **OR 3.95** **(3.77-4.13)** patients 50-59 years vs. patients >80 years.  **OR 4.34** **(4.10-4.60)** patients 40-49 years vs. patients >80 years.  **OR 4.50** **(4.16-4.87)** patients <40 years vs. patients >80 years. | **H** |
| **Johnston et al. 2001**  **Canada** | Retrospective cohort/ 9978 | Adults who died of cancer between 1994 and 1998 in Nova Scotia | | Oncology patient information system and National registries | -Gender  -Community median household income  - Distance from provincial cancer  -Year of death  -Cancer microscopic confirmation  -Cause of death  - Time from diagnosis to death  -Previous palliative radiotherapy | Receipt palliative radiotherapy in the last 9 months of life:   - Receipt palliative radiotherapy consultation with radiation oncologist - Receipt palliative radiotherapy | **- Receipt palliative radiotherapy consultation**  **OR 4.43** **(3.80-5.15)** patients 20-59 years vs. patients >80 years.  **OR 3.76** **(3.25-4.36)** patients 60-69 years vs. patients >80 years.  **OR 2.31** **(2.01-2.65)** patients 70-79 years vs. patients >80 years.  **- Receipt palliative radiotherapy**  **OR 2.09** **(1.63-2.68)** patients 20-59 years vs. patients >80 years.  **OR 1.79** **(1.40-2.27)** patients 60-69 years vs. patients >80 years.  **OR 1.33** **(1.05-1.68)** patients 70-79 years vs. patients >80 years. | **H** |
| **Lavergne et al. 2011**  **Canada** | Retrospective cohort/ 13494 | Adults ≥20 years who died of cancer between 2000 and 2005 in Nova Scotia | | Oncology patient information system and National registries | -Gender  -Major cancer site  -Survival  -Oncology consultation  -Resided in nursing home  -Deprivation  -Travel time | Receipt palliative radiotherapy in the last 9 months of life:   - Receipt palliative radiotherapy consultation with radiation oncologist - Receipt palliative radiotherapy | **- Receipt palliative radiotherapy consultation**  **OR 3.88** **(3.40-4.43)** patients 20-59 years vs. patients >80 years.  **OR 3.08** **(2.71-3.50)** patients 60-69 years vs. patients >80 years.  **OR 2.16** **(1.92-2.43)** patients 70-79 years vs. patients >80 years.  **- Receipt palliative radiotherapy**  **OR 3.88** **(3.35-4.49)** patients 20-59 years vs. patients >80 years.  **OR 3.07 (2.66-3.54)** patients 60-69 years vs. patients >80 years.  **OR 2.08** **(1.81-2.38)** patients 70-79 years vs. patients >80 years. | **H** |
| **Wong et al.**  **2014**  **USA** | Retrospective cohort/ 63221 | Patients diagnosed with lung, breast, prostate and colorectal cancer stage IV between 2000 and 2007 | | Medicare and SEER database (National cancer registry) | -Gender  -Ethnicity/race  -Income  -Year of diagnosis  -Primary tumor  -Geographic region  -Comorbidity  -Survival  -Aggressiveness of end of life care | Receipt palliative radiotherapy | **RR 0.93 (0.91-0.96)** patients 70-74 years vs. patients 66-69 years.  **RR 0.85** **(0.82-0.87)** patients 75-79 years vs. patients 66-69 years.  **RR 0.75** **(0.73-0.78)** patients 80-84 years vs. patients 66-69 years.  **RR 0.56** **(0.53-0.59)** patients ≥85 years vs. patients 66-69 years. | **H** |
| **Adequacy and quality of care** | | | | | | | | |
| **Bergqvist et al. 2022**  **Sweden** | Transversal/ 2516 | Patients >18 years who died of prostate and breast cancer between 2015 and 2019 | National hospital database | | -Socioeconomic status  -Comorbidities  -Diagnosis/gender  -Nursing home resident | Aggressive end of life care:   - Emergency room visits - Hospital admissions | **-Emergency room visits**  **OR 1.50** **(1.12-2.02)** patients 18-64 years vs. patients >85 years.  **OR 1.32** **(1.04-1.67)** patients 65-74 years vs. patients >85 years.  **OR 1.31 (1.04-1.64)** patients 75-84 years vs. patients >85 years. | **H** |
| **Deeb et al.**  **2021**  **USA** | Transversal/ 21335 | Patients >18 years diagnosed with metastatic cancer who died during hospitalization between 1 January 2010 – 31 December 2017 | National inpatient sample database | | -Gender  -Ethnicity /race  -Socioeconomic status  -Health insurance  -Hospital characteristics  -Region of residence | Aggressive care in the last month of life:   - Emergency room visits - Systemic therapy: chemotherapy - Mechanical ventilation | **-Emergency room visits**  **OR 1.14 (1.02-1.27)** patients 50-59 years vs. patients 18-49 years.  OR 0.98 (0.88-1.09) patients 60-69 years vs. patients 18-49 years.  **OR 0.77** **(0.70-0.86)** patients ≥70 years vs. patients 18-49 years.  **-Systemic therapy: chemotherapy**  **OR 0.64 (0.53-0.78)** patients 50-59 years vs. patients 18-49 years.  **OR 0.58** **(0.48-0.70)** patients 60-69 years vs. patients 18-49 years.  **OR 0.27 (0.22-0.34)** patients ≥70 years vs. patients 18-49 years.  **- Mechanical ventilation**  **OR 0.83** **(0.73-0.93)** patients 50-59 years vs. patients 18-49 years.  **OR 0.70 (0.62-0.78)** patients 60-69 years vs. patients 18-49 years.  **OR 0.50 (0.45-0.57)** patients ≥70 years vs. patients 18-49 years. | **H** |
| **Koroukian et al. 2017**  **USA** | Retrospective cohort/ 835 | Patients ≥ 66 years who died of cancer and received their care exclusively through the traditional fee-for-service system | National databases (Medicare and deaths) and national health survey (Health and Retirement Study) | | -Gender  -Race  -Marital status  -Income  -Comorbidity  -Tumor site | Aggressive care in the last month of life:   - Cancer directed treatment with 14 days of death - Acute care visits within 30 days of death | **-Cancer directed treatment with 14 days of death**  OR 1.08 (0.62-1.86) patients 70-74 years vs. patients 66-69 years.  OR 0.99 (0.58-1.69) patients 75-79 years vs. patients 66-69 years.  OR 0.61 (0.34-1.10) patients 80-84 years vs. patients 66-69 years.  **OR 0.45** **(0.23-0.86)** patients >85 years vs. patients 66-69 years.  **-Acute care visits within 30 days of death**  OR 1.06 (0.63-1.77) patients 70-74 years vs. patients 66-69 years.  OR 1.17 (0.70-1.94) patients 75-79 years vs. patients 66-69 years.  OR 0.88 (0.52-1.48) patients 80-84 years vs. patients 66-69 years.  OR 1.08 (0.63-1.86) patients >85 years vs. patients 66-69 years. | **H** |
| **Lindskog et al. 2022**  **Sweden** | Retrospective cohort/ 780 | Patients >18 years diagnosed with malignant brain neoplasm who died between 2015-2019 | Stockholm regional database | | -Gender  -Socioeconomic status  -Comorbidity  -Nursing home resident | Aggressive care in the last month of life:   - Emergency room visits - Hospital admissions | **-Emergency room visits**  OR 0.55 (0.27-1.13) patients 18-64 years vs. patients >85 years.  OR 0.66 (0.32-1.35) patients 65-74 years vs. patients >85 years.  OR 0.72 (0.34-1.50) patients 75-84 years vs. patients >85 years.  **-Hospital admissions**  OR 1.05 (0.51-2.16) patients 18-64 years vs. patients >85 years.  OR 0.95 (0.46-1.96) patients 65-74 years vs. patients >85 years.  OR 1.12 (0.54-2.34) patients 75-84 years vs. patients >85 years. | **H** |
| **Maddison et al. 2012**  **Canada** | Retrospective cohort/ 1201 | Patients ≥ 20 years who died of colorectal cancer between 1 January 2001 and 31 March 2008 | National cancer registry and health and palliative care databases | | -Residence: urban/rural  -Gender  -Income  -Travel distance  -Comorbidity  -Tumor stage  -Long-term care residency  -Emergency room visits in the last month of life  -Place of death  -Year of death | Aggressive care in the last month of life:   - >1 emergency room visits | OR 0.89 (0.6-1.3) patients 65-74 years vs. patients <65 years.  OR 0.70 (0.5-1.1) patients 75-84 years vs. patients <65 years.  OR 0.60 (0.3-1.0) patients ≥85 years vs. patients <65 years. | **H** |
| **Nayar et al.**  **2014**  **USA** | Transversal/  91039 | Medicare beneficiaries ≥ 66 years with lung cancer who died in 2008 | Medicare database | | Not described | Aggressive care in the last 3 months of life:   - Nº emergency room visits - Nº hospital admissions - Nº ICU admissions | **-Nº emergency room visits**  **Regression coefficient -0.12 (*p*=0.0001)** patients >85 years vs. patients 65-74.  **Regression coefficient -0.09 (*p*=0.0001)** patients 75-84 years vs. patients 65-74.  **-Nº hospital admissions**  **Regression coefficient -0.2** **(*p*=0.0001)** patients >85 years vs. patients 65-74.  **Regression coefficient -0.09 (*p*=0.0001)** patients 75-84 years vs. patients 65-74.  **-Nº ICU admissions**  **Regression coefficient -0.52 (*p*=0.0001)** patients >85 years vs. patients 65-74.  **Regression coefficient -0.2** **(*p*=0.0001)** patients 75-84 years vs. patients 65-74. | **M** |
| **Perry et al.**  **2021**  **USA** | Retrospective cohort/ 1290 | Patients who died with metastatic cancer between 1 January 2011 and 31 December 2017 in Louisiana | Health regional database | | -Ethnicity/race  -Gender  -Comorbidity  -Type of cancer | Aggressive care in the last month of life:   - Chemotherapy - Hospital admission - Emergency room visits - ICU admission - Mechanical ventilation | **-Chemotherapy**  **OR 0.76** **(0.69-0.85)** patients >40 years vs. patients <40 years.  **-Hospital admission**  **OR 0.89** **(0.79-0.99)** patients >40 years vs. patients <40 years.  **-Emergency room visits**  OR 1.08 (0.96-1.22) patients >40 years vs. patients <40 years.  **-ICU admission**  OR 0.92 (0.84-1.01) patients >40 years vs. patients <40 years.  **-Mechanical ventilation**  **OR 0.85** **(0.76-0.95)** patients >40 years vs. patients <40 years. | **H** |
| **Ramos-Fernández et al. 2022**  **Puerto Rico** | Retrospective cohort/10755 | Patients ≥ 18 years diagnosed with primary invasive cancer between 2011-2016 and who died during 2011-2017 | Medicare database | | -Gender  -Health insurance  -Health region  -Tumor site  -Comorbidity  -Tumor stage at diagnosis | Aggressive care in the last month of life:   - ≥ 2 emergency room visits | OR 0.95 (0.81-1.12) patients 50-64 years vs. patients <50 years.  **OR 0.71 (0.60-0.86)** patients 65-79 years vs. patients <50 years.  **OR 0.45** **(0.36-0.55)** patients >80 years vs. patients <50 years. | **H** |
| **Tang et al.**  **2012**  **Taiwan** | Retrospective cohort/ 203743 | Adult patients with cancer who died between 2001 and 2006 in Taiwan | National registry of deathls, cancer registry and national health insurance of Taiwan | | -Gender  -Marital status  -Comorbidity  -Cancer diagnosis  -Survival  -Medical speciality  -Hospital charactersitics  -Health care resources | Aggressive care in the last month of life:   - Chemotherapy treatment - >1 emergency room visits - >1 hospital admission - >14 days hospitalization - ICU admission - Intubation - Mechanical ventilation - Cardiopulmonar resuscitation | **- Chemotherapy treatment**  **OR 0.59 (0.57-0.62)** patients 65-74 years vs. patients 18-64 years.  **OR 0.35** **(0.32-0.37)** patients 75-84 years vs. patients 18-64 years.  **OR 0.15** **(0.12-0.17)** patients >85 years vs. patients 18-64 years.  **-> 1 emergency room visits**  **OR 0.90** **(0.88-0.93)** patients 65-74 years vs. patients 18-64 years.  **OR 0.83** **(0.80-0.86)** patients 75-84 years vs. patients 18-64 years.  **OR 0.80** **(0.75-0.85)** patients >85 years vs. patients 18-64 years.  **-> 1 hospital admission**  **OR 0.86** **(0.83-0.89)** patients 65-74 years vs. patients 18-64 years.  **OR 0.80** **(0.77-0.82)** patients 75-84 years vs. patients 18-64 years.  **OR 0.69** **(0.65-0.74)** patients >85 years vs. patients 18-64 years.  **-> 14 days hospitalization**  **OR 0.79** **(0.76-0.81)** patients 65-74 years vs. patients 18-64 years.  **OR 0.75** **(0.72-0.78)** patients 75-84 years vs. patients 18-64 years.  **OR 0.69** **(0.65-0.73)** patients >85 years vs. patients 18-64 years.  **- ICU admission**  **OR 0.86** **(0.80-0.92)** patients 65-74 years vs. patients 18-64 years.  **OR 0.77** **(0.71-0.85)** patients 75-84 years vs. patients 18-64 years.  **OR 0.64** **(0.54-0.75)** patients >85 years vs. patients 18-64 years.  **-Intubation**  **OR 0.92** **(0.88-0.95)** patients 65-74 years vs. patients 18-64 years.  **OR 0.91** **(0.87-0.95)** patients 75-84 years vs. patients 18-64 years.  **OR 0.81** **(0.75-0.88)** patients >85 years vs. patients 18-64 years.  **-Mechanical ventilation**  **OR 0.95** **(0.90-0.99)** patients 65-74 years vs. patients 18-64 years.  **OR 0.93** **(0.88-0.95)** patients 75-84 years vs. patients 18-64 years.  **OR 0.85** **(0.78-0.92)** patients >85 years vs. patients 18-64 years.  **-Cardiopulmonar resuscitation**  **OR 0.91** **(0.88-0.95)** patients 65-74 years vs. patients 18-64 years.  **OR 0.93** **(0.89-0.97)** patients 75-84 years vs. patients 18-64 years.  **OR 0.86** **(0.80-0.92)** patients >85 years vs. patients 18-64 years. | **H** |
| **Watanabe-Galloway et al. 2014**  **USA** | Transversal/ 34975 | Medicare beneficiaries ≥ 66 years with colorectal cancer who died in 2008 | Medicare database | | -Gender  -Ethnicity/race  -Socioeconomic satus  -Comorbidity  -Residence: urban/rural | Aggressive care in the last 3 months of life:   - Nº emergency room visits - Nº hospital admissions - Nº ICU admissions | **-Nº emergency room visits**  **OR 0.93 (*p*=0.0001)** patients >85 years vs. patients 66-74 years.  **OR 0.97 (*p*=0.05)** patients 75-84 years vs. patients 66-74 years.  **-Nº hospital admissions**  **OR 0.83 (*p*=0.0001)** patients >85 years vs. patients 66-74 years.  **OR 0.93 (*p*=0.0001)** patients 75-84 years vs. patients 66-74 years.  **-Nº ICU admissions**  **OR 0.81 (*p*=0.0001)** patients >85 years vs. patients 66-74 years.  OR 1.01 (*NS*) patients 75-84 years vs. patients 66-74 years. | **H** |
| **Palliative care services** | | | | | | | | |
| **Adsersen et al.2021**  **Denmark** | Retrospective cohort/ 5851 | Patients ≥ 18 years diagnosed with pancreatic cancer and who died between 1 July 2011 and 31 December 2018 | Nationa cancer registry and national pallaitive care database | | -Gender  -Atnicancer treatment  -Metastatic cancer  -Year of death  -Comorbidity  -Region of residence | Acces to specialized palliative care | **OR 2.54** **(2.05-3.15)** patients <60 years vs. patients >80 years.  **OR 1.41** **(1.19-1.66)** patients 60-69 years vs. patients >80 years.  **OR 1.29** **(1.11-1.50)** patients 70-79 years vs. patients >80 years. | **H** |
| **Barbera et al. 2010**  **Canada** | Retrospective cohort/ 112398 | Patients who died of cancer in Ontario between 2000 and 2004 | Ontario cancer registry, Ontario Health Insurance Plan, Canadian  Institute for Health Information, Ontario Home Care Administrative System and Registered Persons Database | | -Gender  -Type of cancer  -Comorbidity  -Year of death  -Neighbourhood income  -Rural residence  -Region of residence | Home care:   - Home care in the last 6 months of life - House calls in the last 2 weeks of life | **- Home care in the last 6 months of life**  **OR 0.79 (0.74-0.84)** patients 50-69 years vs. patients <50 years.  **OR 0.56 (0.53-0.59)** patients >70 years vs. patients <50 years.  **-House calls in the last 2 weeks of life**  OR 0.93 (0.87-1.00) patients 50-69 years vs. patients <50 years.  **OR 0.84 (0.79-0.90)** patients >70 years vs. patients <50 years. | **H** |
| **Bergqvist et al. 2022**  **Sweden** | Transversal/ 2516 | Patients >18 years who died of prostate and breast cancer between 2015 and 2019 | National hospital database | | -Socioeconomic status  -Comorbidities  -Diagnosis/gender  -Nursing home resident | Acces to specialized palliative care | **OR 3.31** **(2.32-4.71)** patients 18-64 years vs. patients >85 years.  **OR 2.92** **(2.24-3.82)** patients 65-74 years vs. patients >85 years.  **OR 1.97** **(1.55-2.51)** patients 75-84 years vs. patients >85 years. | **H** |
| **Burge et al. 2008**  **Canada** | Retrospective cohort/ 7511 | Adults who died of cancer between 1 January 1998-31 December 2003 living within 2 District Health Authorities in the province of Nova Scotia, Canada | Administrative health databases and census | | -Gender  -Survival  -Education level  -Income  -Ethnic minority  -Long term care resident  -Urban/rural residence  -Comorbidity  -Cancer cause of death  -Inpatient length of stay  -All physician visits  -At least 1 chemo-related visit  -Radiotherapy received last 6 months of life  -Distance to closest cancer center | Registration with a palliative care program during the last 6 mothns of life | **OR 0.80** **(0.7-0.9)** patients 65-74 years vs. patients <65 years.  **OR 0.70** **(0.6-0.8)** patients 75-84 years vs. patients <65 years.  **OR 0.40** **(0.3-0.5)** patients >85 years vs. patients <65 years. | **H** |
| **Craigs et al. 2018**  **UK** | Retrospective cohort/ 2474 | Patients > 18 years who died of cancer between January 2010-February 2012 | Northern and  Yorkshire Cancer Registry, medical records and a clinical information system | | Not described | Community and/or hospital palliative care provision | **-Community palliative care**  OR 1.08 (0.60-1.97) patients <50 years vs. patients >80 years.  OR 1.24 (0.80-1.93) patients 50-59 years vs. patients >80 years.  OR 1.21 (0.87-1.68) patients 60-69 years vs. patients >80 years.  OR 1.14 (0.86-1.51) patients 70-79 years vs. patients >80 years.  **-Hospital palliative care**  **OR 2.12 (1.18-3.84)** patients <50 years vs. patients >80 years.  **OR 1.96 (1.25-3.07)** patients 50-59 years vs. patients >80 years.  **OR 1.66 (1.17-2.35)** patients 60-69 years vs. patients >80 years.  **OR 1.37 (1.01-1.86)** patients 70-79 years vs. patients >80 years.  **-Community and hospital palliative care**  **OR 2.55 (1.43-4.54)** patients <50 years vs. patients >80 years.  **OR 2.23 (1.43-3.49)** patients 50-59 years vs. patients >80 years.  **OR 1.75 (1.24-2.48)** patients 60-69 years vs. patients >80 years.  OR 1.35 (0.99-1.83) patients 70-79 years vs. patients >80 years. | **H** |
| **Han et al. 2021**  **USA** | Transversal/ 131852 | Patients ≥ 18 years hospitalized with metastatic bladder cancer between 2003 and 2014 | National Inpatient Sample database | | -Gender  -Race  -Income  -Comorbidity  -Hospital characteristics  -Cancer related factors  -Treatment factors | Inpatient palliative care | **OR 1.02** **(1.01-1.02)** older patients vs. younger patients. | **H** |
| **Hegagi et al. 2022**  **USA** | Retrospective cohort/ 6888 | Patients diagnosed with pancreatic cancer between 1 April 2010 and 31 March 2016 | Ontatario cancer registry , home care database and registered person database | | -Gender  -Rurality  -Income  -Cancer stage  -Comorbidity | Home care (speciallized pallative care units) | OR 1.19 (0.80-1.77) patients 45-54 years vs. patients 18-44 years.  **OR 1.54** **(1.06-2.23)** patients 55-64 years vs. patients 18-44 years.  **OR 1.72** **(1.18-2.49)** patients 65-74 years vs. patients 18-44 years.  **OR 2.30** **(1.58-3.35)** patients 75-84 years vs. patients 18-44 years.  **OR 3.07** **(2.03-4.65)** patients >85 years vs. patients 18-44 years. | **H** |
| **Heller et al. 2019**  **USA** | Retrospective cohort/ 86573 | Patients ≥ 65 with colorectal cancer requiring emergent surgery between 2009-2014 | National Inpatient Sample database | | -Gender  -Race  -Comorbidity  -Metastasic disease  -Income  -Discharge year  -Hospital bedsize  -Hospital region  -Hospital control  -Teaching hospital  -Urbanicity | Inpatient palliative care consultation | **OR 1.4 (1.1-1.9)** patients 70-74 years vs. patients 65-69 years.  **OR 1.4 (1.1-1.9)** patients 74-79 years vs. patients 65-69 years.  **OR 1.6** **(1.2-2.1)** patients 80-84 years vs. patients 65-69 years.  **OR 2.3** **(1.7-3.0)** patients 85-89 years vs. patients 65-69 years.  **OR 3.3** **(2.4-4.5)** patients >90 years vs. patients 65-69 years. | **H** |
| **Hunt et al.**  **2002**  **Australia** | Transversal/ 3086 | Patients who died of cancer in 1999 in South Australia | State cancer registry, death registry and the Australian Institute of Health and  Welfare. | | -Residence  -Country of birth  -Primary cancer site  -Survival | Use of palliative care services (hospice care) | OR 0.96 (0.72-1.27) patients 60-69 years vs. patients <60 years.  OR 0.83 (0.64-1.07) patients 70-79 years vs. patients <60 years.  **OR 0.50 (0.39-0.65)** patients >80 years vs. patients <60 years. | **H** |
| **Jackson et al. 2022**  **USA** | Retrospective cohort/ 23567 | Patients ≥ 18 years hospitalized with esophageal cancer between 2016-2018 | National Inpatient Sample database | | -Income  -Gender  -Ethnicity/race  -Health insurance  -Hospital characteristics  -Comorbidity  -Length of stay  -Chemotherapy  -Admission type  -Patient disposition | Inpatient palliative care | OR 1.08 (0.96-1.22) patients >65 years vs. patients <65 years | **H** |
| **Koroukian et al.**  **2017**  **USA** | Retrospective cohort/ 835 | Patients ≥ 66 years who died of cancer and received their care exclusively through the traditional fee-for-service system | National databases (Medicare and deaths) and national health survey (Health and Retirement Study) | | -Gender  -Race  -Marital status  -Income  -Comorbidity  -Tumor site | Hospice care | OR 0.91 (0.54-1.56) patients 70-74 years vs. patients 66-69 years.  OR 0.78 (0.47-1.32) patients 75-79 years vs. patients 66-69 years.  OR 1.01 (0.59-1.74) patients 80-84 years vs. patients 66-69 years.  OR 1.15 (0.65-2.02) patients >85 years vs. patients 66-69 years. | **H** |
| **Lackan et al. 2003**  **USA** | Retrospective cohort/ 25161 | Woman ≥ 65 years diagnosed with breast cancer between 1986-1996 and who died between 1991 -1996 | Medicare and SEER database (National cancer registry) | | Not described | Hospice care | Patients 65-74 years vs. patients > 90 years **(25.4% vs. 12.3%;** ***p*<0.001)**.  Patients 75-84 years vs. patients > 90 years **(22.8% vs. 12.3%;** ***p*<0.001**).  Patients 85-89 years vs. patients > 90 years **(18.1% vs. 12.3%;** ***p*<0.001)**. | **M** |
| **Lai et al. 2020**  **Taiwan** | Retrospective cohort/ 516409 | Patients >18 years diagnosed with cancer and who died between 1 January 2006 -31 Decembre 2016 | National Health Insurance  Research Database | | -Gender  -Comorbidity  -Socioeconomic status | Hospice care | **OR 0.73** **(0.72-0.74)** patients >65 years vs. patients 18-64 years. | **H** |
| **Lee et al. 2021**  **USA** | Retrospective cohort/ 204175 | Patients >18 years with advanced cancer hospitalized between 2012-2014 | National Inpatient Sample database | | -Gender  -Race  -Socioeconomic status  -Comorbidity  -Surgical procedure  -Chemotherapy or radiotherpay  -Gastrostomy tube  -Parenteral nutrition  -Hemodialysis  -Posoperative complications  -Length of stay  -Hospital characteristics | Inpatient palliative care consultation | **OR 1.01** **(1.006-1.009)** older patients vs. younger patients | **H** |
| **Lindskog et al. 2022**  **Sweden** | Retrospective cohort/ 780 | Patients >18 years diagnosed with malignant brain neoplasm who died between 2015-2019 | Stockholm regional database | | -Gender  -Socioeconomic status  -Comorbidity  -Nursing home resident | Access to specialized palliative care during the last 3 months of life | **OR 3.03** **(1.37-6.70)** patients 18-64 years vs. patients >85 years.  **OR 2.70** **(1.23-5.93)** patients 65-74 years vs. patients >85 years.  **OR 2.50** **(1.11-5.66)** patients 75-84 years vs. patients >85 years. | **H** |
| **Maddison et al. 2012**  **Canada** | Retrospective cohort/ 1201 | Patients ≥ 20 years who died of colorectal cancer between 1 January 2001 and 31 March 2008 | National cancer registry and health and palliative care databases | | -Residence: urban/rural  -Gender  -Income  -Travel distance  -Comorbidity  -Tumor stage  -Long-term care residency  -Emergency room visits in the last month of life  -Place of death  -Year of death | Access to a palliative care program:   - Registration in a palliative care program - Registration ≥60 days prior to death | **-Registration in a palliative care program**  OR 0.79 (0.4-1.5) patients 65-74 years vs. patients <65 years.  OR 0.69 (0.4-1.3) patients 75-84 years vs. patients <65 years.  **OR 0.32** **(0.2-0.7)** patients ≥85 years vs. patients <65 years.  **-Registration ≥60 days prior to death**  OR 0.88 (0.5-1.5) patients 65-74 years vs. patients <65 years.  **OR 0.51** **(0.3-0.9)** patients entre 75-84 years vs. patients <65 years.  OR 0.51 (0.2-1.2) patients ≥85 years vs. patients <65 years. | **H** |
| **Milki et al.**  **2021**  **USA** | Retrospective cohort/ 4559 | Patients ≥ 18 years hospitalized who died between 2005-2011 with gyneacological cancer | National Inpatient Sample database | | -Time period  -Race  -Cancer type  -Socioeconomic status  -Health insurance  -Hospital characteristics  -Length of stay  -Severity of illness  -Depression  -Residing where physician-assisted death is legal | Inpatient palliative care | **OR 1.36 (1.11-1.68)** patients >65 years vs. patients <65 years. | **H** |
| **Nayar et al.**  **2014**  **USA** | Transversal/  91039 | Medicare beneficiaries ≥ 66 years with lung cancer who died in 2008 | Medicare database | | Not described | Access to hospice care:   - Hospice care use - Hospice enrollment in last 3 days | **-Hospice care use**  **OR 1.29** **(*p*=0.0001)** patients >85 years vs. patients entre 65-74.  **OR 1.17** **(*p*=0.0001)** patients 75-84 years vs. patients entre 65-74.  **-Hospice enrollment in last 3 days**  **OR 0.77** **(*p*=0.0001)** patients 75-84 years vs. patients entre 65-74.  **OR 0.87** **(*p*=0.0001)** patients 75-84 years vs. patients entre 65-74. | **M** |
| **Rosenfeld et al. 2018**  **USA** | Retrospective cohort/ 67947 | Patients ≥ 18 years hospitalized with metastasic gyneacological cancer (ovarian, uterine and cervical) between 2005 and 2011 | National Inpatient Sample database | | -Race  -Socioeconomic status  -Cancer type  -Health insurance  -Survival  -Hospital location  -Hospital region  -Hospital bedsize  -Hospital teaching status | Inpatient palliative care | **OR 1.52** **(1.36-1.70)** patients >63 years vs. younger patients. | **H** |
| **Rubens et al. 2019**  **USA** | Retrospective cohort/ 4732172 | Patients ≥18 years hospitalized with advanced cancer between 2005-2014 | National Inpatient Sample database | | -Gender  -Race  -Socioeconomic status  -Cancer type  -Health insurance  -Hospital region  -Hospital bedsize  -Hospital teaching status  -Do not resuscitate status  -Previous radiotherapy  -Chemotherapy, mechanical ventilation, parenteral o enteral nutrition, hemodialysis | Inpatient palliative care | **OR 1.05** **(1.01-1.08)** patients 60-69 years vs. patients <60 years.  **OR 1.15** **(1.09-1.21)** patients 70-79 years vs. patients <60 years.  **OR 1.47** **(1.38-1.56)** patients >80 years vs. patients <60 years. | **H** |
| **Sharp et al.**  **2018**  **Sweden** | Transversal/ 1872 | Patients ≥18 years diagnosed between 2014-2016 with primary gyneacological, hematological, head and neck and upper gastrointestinal cancer, living within the Stockholm Gotland area | Questionnaire | | -Gender  -Cancer type  -Place of birth  -Occupation status  -Education level  -Living situation  -Treatment and type of treatment | Acceso to supportive care:   - Contact nurse - Individual written plans - Patient advocacy groups - Referred to palliative care | **-Contact nurse**  OR 0.99 (0.97-1.0) older patients vs. younger patients.  **-Individual written plans**  OR 0.99 (0.98-1.01) older patients vs. younger patients.  **-Patient advocacy groups**  **OR 0.98** **(0.96-0.99)** older patients vs. younger patients.  **-Referred to palliative care**  OR 0.99 (0.98-1.01) older patients vs. younger patients. | **H** |
| **Tang et al.**  **2012**  **Taiwan** | Retrospective cohort/ 203743 | Adult patients with cancer who died between 2001 and 2006 in Taiwan | National registry of deathls, cancer registry and national health insurance of Taiwan | | -Gender  -Marital status  -Comorbidity  -Cancer diagnosis  -Survival  -Medical speciality  -Hospital charactersitics  -Health care resources | Acces and use of palliative care during the last month of life:   - Use of hospice services - Referral to hospice services in the last three days of life. | **-Use of hospice services**  **OR 1.08** **(1.03-1.14)** patients 65-74 years vs. patients 18-64 years.  **OR 1.14 (1.07-1.22)** patients 75-84 years vs. patients 18-64 years.  **OR 1.10** **(1.01-1.20)** patients >85 years vs. patients 18-64 years.  **-Referral to hospice services in the last three days of life**  OR 1.00 (0.95-1.06) patients 65-74 years vs. patients 18-64 years.  OR 0.94 (0.85-1.03) patients 75-84 years vs. patients 18-64 years.  **OR 0.87** **(0.75-0.99)** patients >85 years vs. patients 18-64 years. | **H** |
| **Watanabe-Galloway et al. 2014**  **USA** | Transversal/ 34975 | Medicare beneficiaries ≥ 66 years with colorectal cancer who died in 2008 | Medicare database | | -Gender  -Ethnicity/race  -Socioeconomic satus  -Comorbidity  -Residence: urban/rural | Access to hospice care:   - Hospice care use - Hospice enrollment in last 3 days | **-Hospice care use**  OR 0.94 (*NS*) patients ≥85 years vs. patients 66-74 years.  **OR 0.93 (*p*=0.0001)** patients 75-84 years vs. patients 66-74 years.  **-Hospice enrollment in last 3 days**  OR 0.93 (*NS*) patients ≥85 years vs. patients entre 66-74 years.  OR 0.95 (*NS*) patients 75-84 years vs. patients 66-74 years. | **H** |
| **Advance care planning** | | | | | | | | |
| **Saeed et al. 2018**  **USA** | Transversal/ 383 | Patients ≥ 21 years with advanced cancer (stage IV cancer non-hematologic cancer or stage III cancer) | Survey | | -Race  -Health insurance  -Perceibed financial strain  -Cancer type  -Marital status  -Living situation  -Quality of life (MQOL)  -Distress  -PEACE scale  -Physical and social well-being scale (FACT-G)  -Presence or absence of caregiver  -Study site | Preference for palliative care | **OR 0.49** **(0.28-0.88)** patients >65 years vs. patients <65 years. | **M** |
| **Saeed et al. 2019**  **USA** | Transversal/ 265 | Patients ≥ 21 years with advanced cancer (stage IV cancer non-hematologic cancer or stage III cancer) | Survey | | -Gender  -Race  -Perceibed financial strain  -Income  -Education level  -Doctor’s subspecialty  -Study site | Completion of advance directives | **Regression coefficient 0.01** **(0.01-0.02)** older patients vs. younger patients | **M** |
| **Place of death** | | | | | | | | |
| **Barbera et al. 2010**  **Canada** | Retrospective cohort/ 112398 | Patients who died of cancer in Ontario between 2000 and 2004 | Ontario cancer registry, Ontario Health Insurance Plan, Canadian  Institute for Health Information, Ontario Home Care Administrative System and Registered Persons Database | | -Gender  -Type of cancer  -Comorbidity  -Year of death  -Neighbourhood income  -Rural residence  -Region of residence | Acute care hospital death | **OR 0.88 (0.83-0.94)** patients 50-69 years vs. patients <50 years.  **OR 0.69 (0.65-0.73)** patients >70 years vs. patients <50 years. | **H** |
| **Bergqvist et al. 2022**  **Sweden** | Transversal/ 2516 | Patients >18 years who died of prostate and breast cancer between 2015 and 2019 | National hospital database | | -Socioeconomic status  -Comorbidities  -Diagnosis/gender  -Nursing home resident | Acute care hospital death | **OR 2.88** **(1.87-4.43)** patients 18-64 years vs. patients >85 years.  **OR 2.09** **(1.44-3.03)** patients 65-74 years vs. patients >85 years.  **OR 1.81** **(1.28-2.58)** patients 75-84 years vs. patients >85 years. | **H** |
| **Burge et al.**  **2005**  **Canada** | Retrospective cohort/ 13652 | Adult patients residing in Nova Scotia and who died of cancer between 1992-1997 | Administrative health databases and census | | -Gender  -Cancer type  -Income  -Year of death  -Survival  -Received a visit in long term-care  -Lengh of stay | Out of hospital death | OR 0.91 (0.71-1.17) patients 45-64 years vs. patients 18-45 years.  OR 0.92 (0.71-1.18) patients 65-74 years vs. patients 18-45 years.  OR 0.96 (0.75-1.23) patients 75-85 years vs. patients 18-45 years.  OR 1.07 (0.82-1.40) patients >85 years vs. patients 18-45 years. | **H** |
| **D’Angelo et al. 2020**  **Italy** | Retrospective cohort/ 13656 | Patients > 18 years residents in Lazio region who died of advanced cancer between January 2012-December 2016 | Hospital discharge register and electronic medical records | | -Gender  -Ethnicity  -Education level  -Citizenship  -Area of residence  -Distance to closest specialised palliative service  -Cancer type  -Survival | Home or hospice care facility death vs. hospital death | OR 1.04 (0.87-1.25) patients 67-75 years vs. patients ≤66 years.  **OR 1.45** **(1.21-1.73)** patients 76-82 years vs. patients ≤66 years.  **OR 1.81** **(1.52-2.16)** patients ≥83 years vs. patients ≤66 years. | **H** |
| **Gatrell et al. 2003**  **UK** | Transversal/ 6900 | Patients who died of cancer between 1993-2000 | National registers | | Not described | - Home - Acute care hospital - Hospice - Nursing home | **-Home**  **OR 0.979 (0.974-0.983)** older patients vs. younger patients.  **-Acute care hospital**  **OR 1.015** **(1.010-1.019)** older patients vs. younger patients.  **-Hospice**  **OR 0.975** **(0.971-0.980)** older patients vs. younger patients.  **-Nursing home**  **OR 1.115 (1.103-1.127)** older patients vs. younger patients. | **M** |
| **Hegagi et al.**  **2022**  **USA** | Retrospective cohort/ 6888 | Patients diagnosed with pancreatic cancer between 1 Abpril 2010- 31 March 2016 | National cancer registry and home care database | | -Gender  -Rurality  -Income  -Cancer stage  -Comorbidity  -Home care category | Out of hospital death | OR 1.15 (0.72-1.84) patients 45-54 years vs. patients 18-44 years.  OR 1.08 (0.69-1.70) patients 55-64 years vs. patients 18-44 years.  OR 0.99 (0.63-1.55) patients 65-74 years vs. patients 18-44 years.  OR 0.95 (0.60-1.50) patients 75-84 years vs. patients 18-44 years.  OR 0.98 (0.59-1.65) patients >85 years vs. patients 18-44 years. | **H** |
| **Hunt et al.**  **2001**  **Australia** | Transversal/ 29230 | Patients who died of cancer in South Australia between 1990-1999 | State cancer registry | | -Gender  -Race  -Country of birth  ­-Place of residence  -Socieconomic status  -Survival  -Year of death  -Cancer type | - Metropolitan private hospital - Country hospital - Hospice - Nursing home - Home - Metropolitan public hospital | **Place of death vs. metropolitan public hospital**  **-Metropolitan private hospital**  **OR 1.44 (1.26-1.65)** patients 60-69 years vs. patients <60 years.  **OR 1.50 (1.32-1.70)** patients 70-79 years vs. patients <60 years.  **OR 2.01 (1.75-2.31)** patients >80 years vs. patients <60 years.  **-Country hospital**  **OR 1.52 (1.31-1.76)** patients 60-69 years vs. patients <60 years.  **OR 1.62 (1.40-1.86)** patients 70-79 years vs. patients <60 years.  **OR 2.26 (1.93-2.65)** patients >80 years vs. patients <60 years.  **-Hospice**  OR 1.06 (0.95-1.18) patients 60-69 years vs. patients <60 years.  **OR 1.21 (1.09-1.34)** patients 70-79 years vs. patients <60 years.  **OR 1.17 (1.04-1.32)** patients >80 years vs. patients <60 years.  -**Nursing home**  **OR 3.30 (2.45-4.46)** patients 60-69 years vs. patients <60 years.  **OR 9.70 (7.36-12.78)** patients 70-79 years vs. patients <60 years.  **OR 36.24 (27.53-47.71)** patients >80 years vs. patients <60 years.  **-Home**  OR 0.99 (0.88-1.11) patients 60-69 years vs. patients <60 years.  **OR 0.86 (0.77-0.96)** patients 70-79 years vs. patients <60 years.  **OR 0.85 (0.75-0.96)** patients >80 years vs. patients <60 years. | **H** |
| **Koroukian et al. 2017**  **USA** | Retrospective cohort/ 835 | Patients ≥ 66 years who died of cancer and received their care exclusively through the traditional fee-for-service system | National databases (Medicare and deaths) and national health survey (Health and Retirement Study) | | -Gender  -Race  -Marital status  -Income  -Comorbidity  -Tumor site | In hospital death | OR 1.27 (0.69-2.37) patients 70-74 years vs. patients 66-69 years.  OR 1.20 (0.65-2.19) patients 75-79 years vs. patients 66-69 years.  OR 1.00 (0.53-1.91) patients 80-84 years vs. patients 66-69 years.  OR 0.62 (0.31-1.23) patients >85 years vs. patients 66-69 years. | **H** |
| **Lindskog et al. 2022**  **Sweden** | Retrospective cohort/ 780 | Patients >18 years diagnosed with malignant brain neoplasm who died between 2015-2019 | Stockholm regional database | | -Gender  -Socioeconomic status  -Comorbidity  -Nursing home resident | Acute care hospital death | OR 2.53 (0.57-11.12) patients 18-64 years vs. patients >85 years.  OR 1.80 (0.41-8.00) patients 65-74 years vs. patients >85 years.  OR 1.16 (0.25-5.39) patients 75-84 years vs. patients >85 years. | **H** |
| **Maddison et al. 2012**  **Canada** | Retrospective cohort/ 1201 | Patients ≥ 20 years who died of colorectal cancer between 1 January 2001 and 31 March 2008 | National cancer registry and health and palliative care databases | | -Residence: urban/rural  -Gender  -Income  -Travel distance  -Comorbidity  -Tumor stage  -Long-term care residency  -Emergency room visits in the last month of life  -Place of death  -Year of death | In hospital death | OR 1.15 (0.8-1.8) patients 65-74 years vs. patients <65 years.  OR 0.85 (0.6-1.2) patients 75-84 years vs. patients <65 years.  OR 0.94 (0.6-1.5) patients ≥85 years vs. patients <65 years. | **H** |
| **Neergaard et al. 2012**  **Denmark** | Transversal/ 599 | Adult patients living in Aarhus (Denmark) who died of cancer between 1 March and 30 November 2006 | National registers | | -Gender  -Diagnosis  -Marital status  -Children residing at home  -Housing space  -Inmigrant  -Conctact with general practitioner  -Lengh of hospital stay  - Specialist team involvement | Home death | **PR 0.83 (0.70-0.99)** patients 70-79 years vs. patients >80 years.  PR 0.82 (0.66-1.01) patients 60-69 years vs. patients >80 years.  **PR 0.67 (0.45-0.99)** patients 50-59 years vs. patients >80 years.  PR 0.67 (0.39-1.15) patients 18-49 years vs. patients >80 years. | **H** |
| **Öhlén et al. 2017**  **Sweden** | Transversal/ 20710 | Patients who died of cancer in 2012, Sweden | National registers | | -Gender  -Cancer type  -Marital status  -Educational level  -Urbanicity  -Health care region | - Home - Hospital - Nursing home | **-Hospital vs. home (place of residence)**  OR 1.13 (0.88-1.46) patients 18-49 years vs. patients 90-95+.  OR 1.19 (0.96-1.47) patients 50-59 years vs. patients 90-95+.  **OR 1.31 (1.09-1.57)** patients 60-69 years vs. patients 90-95+.  **OR 1.28 (1.07-1.52)** patients 70-79 years vs. patients 90-95+.  **OR 1.30 (1.09-1.54)** patients 80-89 years vs. patients 90-95+.  **-Nursing home vs. home (place of residence)**  **OR 0.12 (0.08-0.17)** patients 18-49 years vs. patients 90-95+.  **OR 0.19 (0.15-0.24)** patients 50-59 years vs. patients 90-95+.  **OR 0.25 (0.20-0.30)** patients 60-69 years vs. patients 90-95+.  **OR 0.42 (0.35-0.50)** patients 70-79 years vs. patients 90-95+.  **OR 0.78 (0.66-0.93)** patients 80-89 years vs. patients 90-95+.  **-Hospital vs. nursing home (place of residence)**  OR 0.77 (0.28-2.12) patients 60-69 years vs. patients 90-95+.  OR 0.66 (0.31-1.43) patients 70-79 years vs. patients 90-95+.  OR 1.54 (0.88-2.67) patients 80-89 years vs. patients 90-95+. | **H** |
| **Papke et al.**  **2007**  **Germany** | Transversal/ 2316 | Patients who died of cancer between 1997-2003 | Health Office of the administrative district “*Sächische Schweiz*” | | Not described | Nursing home | **OR 0.30 (0.14-0.67)** patients 61-70 years vs. patients <40 years.  **OR 0.40 (0.26-0.61)** patients 71-80 years vs. patients <40 years. | **M** |
| **Sedhom et al. 2021**  **USA** | Retrospective cohort/ 3182707 | Patients >65 years who died of lung, colorectal, prostate, pancreatic and breast cancer between 2003 -2017 | National register | | -Gender  -Year of death  -Ethnicity/race  -Primary cancer diagnosis | - Hospice facility - Hospital - Nursing home - Home | **Place of death vs. home**  **-Hospice facility**  **OR 0.98 (0.97-0.99)** patients 70-74 years vs. patients 65-69 years.  **OR 0.96 (0.95-0.97)** patients 75-79 years vs. patients 65-69 years.  **OR 0.98 (0.97-0.99)** patients 80-84 years vs patients 65-69 years.  OR 0.99 (0.98-1.01) patients 85-89 years vs. patients 65-69 years.  **OR 0.98 (0.96-0.99)** patients 90-94 years vs. patients 65-69 years.  **OR 0.92 (0.89-0.96)** patients 95-99 years vs. patients 65-69 years.  **OR 0.73 (0.66-0.81)** patients >100 years vs. patients 65-69 years.  **-Hospital**  **OR 0.93 (0.92-0.94)** patients 70-74 years vs. patients 65-69 years.  **OR 0.87 (0.86-0.87)** patients 75-79 years vs patients 65-69 years.  **OR 0.77 (0.77-0.78)** patients 80-84 years vs. patients 65-69 years.  **OR 0.71 (0.70-0.71)** patients 85-89 years vs. patients 65-69 years.  **OR 0.63 (0.62-0.64)** patients 90-94 years vs. patients 65-69 years.  **OR 0.59 (0.58-0.61)** patients 95-99 years vs. patients 65-69 years.  **OR 0.51 (0.47-0.55)** patients >100 years vs patients 65-69 years.  **-Nursing home**  **OR 1.17 (1.16-1.19)** patients 70-74 years vs. patients 65-69 years.  **OR 1.48 (1.46-1.50)** patients 75-79 years vs. patients 65-69 years.  **OR 1.97 (1.94-1.99)** patients 80-84 years vs. patients 65-69 years.  **OR 2.75 (2.72-2.78)** patients 85-89 years vs. patients 65-69 years.  **OR 3.65 (3.60-3.70)** patients 90-94 years vs. patients 65-69 years.  **OR 4.69 (4.59-4.79)** patients 95-99 years vs. patients 65-69 years.  **OR 5.02 (4.76-5.29)** patients >100 years vs. patients 65-69 years. | **H** |
| **Tang et al.**  **2012**  **Taiwan** | Retrospective cohort/ 203743 | Adult patients with cancer who died between 2001 and 2006 in Taiwan | National registry of deathls, cancer registry and national health insurance of Taiwan | | -Gender  -Marital status  -Comorbidity  -Cancer diagnosis  -Survival  -Medical speciality  -Hospital charactersitics  -Health care resources | Acute care death | **OR 0.83** **(0.75-0.87)** patients 65-74 years vs. patients 18-64 years.  **OR 0.84** **(0.80-0.88)** patients 75-84 years vs. patients 18-64 years.  **OR 0.83** **(0.78-0.89)** patients >85 years vs. patients 18-64 years. | **H** |

NS: Not signifiant; USA: Unit States of America; ICU: Intensive Care Unit; OR: *odds ratio*; RR: relative risk; CI: confidence interval

*Statistically significant results highlighted in bold (p<0.05).

§Results of the evaluation of methodological quality using the NewCastle-Ottawa Scale (NOS).Categorized in high (H), moderate (M) and low (L).

Table 7. Characteristics of the studies analysing social inequalities bye GENDER and palliative care approaches

| **Author/ Year/Country** | **Study design/ N** | **Study population** | **Collection method (extraction of information)** | **Adjustment variables** | **Outcome measure** | **Main results** | **NOS^§^** |
| --- | --- | --- | --- | --- | --- | --- | --- |
| **Symptom management** | | | | | | | |
| **Åsli et al. 2018**  **Norway** | Retrospective cohort/ 25287 | Patients who died with cancer (excluding non-melanoma skin cancer) between 1 July 2009 -31 December 2011 in Norway | National registry | -Age  -Cancer site  -Survival  -County of residence  -Previous curative radiotherapy  -Education level  -Household income  -Radiotherapy facility at diagnosing hospital  -Travel distance | Receipt of palliative radiotherapy in the last two years of life | **OR 0.92** **(0.85-0.99)** woman vs. man. | **H** |
| **Colibaseanu et al. 2018**  **USA** | Retrospective cohort/ 287923 | Patients who died of colorectal cancer between 2004 and 2013 | National cancer registry | -Year of diagnosis  -Age  -Ethnicity/race  -Health insurance  -Income  -Education level  -Urban/rural residence  -Travel distances  -Comorbidities  -Hospital characteristics  -Hospital region  -Percentage of patients receiving palliative care  -Tumor characteristics | Receipt of surgery, radiotherapy, chemotherapy with palliative intent and/or pain management | ***Survival <6 months***  OR 0.95 (0.9-1.01) man vs. woman.  ***Survival 6-24 months***  OR 1.02 (0.96-1.09) man vs. woman.  ***Survival >24 months***  OR 0.97 (0.87-1.07) man vs. woman. | **H** |
| **Dumbrava et al. 2018**  **Australia** | Retrospective cohort/ 1173 | Patients diagnosed with unresectable pancreatic adenocarcinoma between July 2009 and June  2011, residents in Queensland and New South Wales, Australia | Medical records and National cancer registry | -Age  -Charlson comorbidity index  -ECOG scale | NOT receipt palliative chemotherapy during the first year following diagnosis | OR 1.24 (0.95-1.62) woman vs. man. | **H** |
| **Johnston et al. 2001**  **Canada** | Retrospective cohort/ 9978 | Adults who died of cancer between 1994 and 1998 in Nova Scotia | Oncology patient information system and National registries | -Age  -Community median household income  - Distance from provincial cancer  -Year of death  -Cancer microscopic confirmation  -Cause of death  - Time from diagnosis to death  -Previous palliative radiotherapy | Receipt palliative radiotherapy in the last 9 months of life:   - Receipt palliative radiotherapy consultation with radiation oncologist - Receipt palliative radiotherapy | **-Receipt palliative radiotherapy consultation**  OR 1.04 (0.93-1.15) man vs. woman.  **-Receipt palliative radiotherapy**  OR 0.95 (0.81-1.12) man vs. woman. | **H** |
| **Lavergne et al. 2011**  **Canada** | Retrospective cohort/ 13494 | Adults ≥20 years who died of cancer between 2000 and 2005 in Nova Scotia | Oncology patient information system and National registries | -Age  -Major cancer site  -Survival  -Oncology consultation  -Resided in nursing home  -Deprivation  -Travel time | Receipt palliative radiotherapy in the last 9 months of life:   - Receipt palliative radiotherapy consultation with radiation oncologist - Receipt palliative radiotherapy | **-Receipt palliative radiotherapy consultation**  **OR 1.19** **(1.09-1.30)** man vs. woman.  **-Receipt palliative radiotherapy**  **OR 1.18** **(1.06-1.30)** man vs. woman. | **H** |
| **Adequacy and quality of care** | | | | | | | |
| **Bergqvist et al. 2022**  **Sweden** | Transversal/ 2516 | Patients >18 years who died of prostate and breast cancer between 2015 and 2019 | National hospital database | -Age  -Socioeconomic status  -Comorbidities  -Nursing home resident | Aggressive end of life care:   - Emergency room visits - Hospital admissions | **-Hospital admissions**  **OR 1.31** **(1.10-1.57)** woman vs. man. | **H** |
| **Deeb et al. 2021**  **USA** | Transversal/ 21335 | Patients >18 years diagnosed with metastatic cancer who died during hospitalization between 1 January 2010 – 31 December 2017 | National inpatient sample database | -Age  -Ethnicity /race  -Socioeconomic status  -Health insurance  -Hospital characteristics  -Region of residence | Aggressive care in the last month of life:   - Emergency room visits - Systemic therapy: chemotherapy - Mechanical ventilation | **-Emergency room visits**  OR 1.01 (0.95-1.08) woman vs. man.  **-Systemic therapy: chemotherapy**  **OR 1.18 (1.03-1.36)** woman vs. man.  **-Mechanical ventilation**  OR 0.89 (0.83-0.96) woman vs. man. | **H** |
| **Koroukian et al. 2017**  **USA** | Retrospective cohort/ 835 | Patients ≥ 66 years who died of cancer and received their care exclusively through the traditional fee-for-service system | National databases (Medicare and deaths) and national health survey (Health and Retirement Study) | -Age  -Race  -Marital status  -Income  -Comorbidity  -Tumor site | Aggressive care in the last month of life:   - Cancer directed treatment with 14 days of death - Acute care visits within 30 days of death | **-Cancer directed treatment with 14 days of death**  OR 0.99 (0.68-1.44) woman vs. man.  **-Acute care visits within 30 days of death**  OR 0.84 (0.61-1.16) woman vs. man. | **H** |
| **Lindskog et al. 2022**  **Sweden** | Retrospective cohort/ 780 | Patients >18 years diagnosed with malignant brain neoplasm who died between 2015-2019 | Stockholm regional database | -Age  -Socioeconomic status  -Comorbidity  -Nursing home resident | Aggressive care in the last month of life:   - Emergency room visits - Hospital admissions | **-Emergency room visits**  OR 0.83 (0.59-1.15) woman vs. man.  **-Hospital admissions**  **OR 0.72** (0.53-0.99) woman vs. man. | **H** |
| **Maddison et al. 2012**  **Canada** | Retrospective cohort/ 1201 | Patients ≥ 20 years who died of colorectal cancer between 1 January 2001 and 31 March 2008 | National cancer registry and health and palliative care databases | -Residence: urban/rural  -Age  -Income  -Travel distance  -Comorbidity  -Tumor stage  -Long-term care residency  -Emergency room visits in the last month of life  -Place of death  -Year of death | Aggressive care in the last month of life:   - >1 emergency room visits | OR 0.70 (0.6-1.0) woman vs. man. | **H** |
| **Nayar et al. 2014**  **USA** | Transversal/ 91039 | Medicare beneficiaries ≥ 66 years with lung cancer who died in 2008 | Medicare database | Not described | Aggressive care in the last 3 months of life:   - Nº emergency room visits - Nº hospital admissions - Nº ICU admissions | **-Nº hospital admissions**  **Regression coefficient -0.01 (*p*=0.05)** woman vs. man.  **-Nº emergency room visits**  Regression coefficient -0.003 (*NS*) woman vs. man.  **-Nº ICU admissions**  **Regression coefficient -0.13 (*p*=0.0001)** woman vs. man. | **M** |
| **Perry et al. 2021**  **USA** | Retrospective cohort/ 1290 | Patients who died with metastatic cancer between 1 January 2011 and 31 December 2017 in Louisiana | Health regional database | -Ethnicity/race  -Age  -Comorbidity  -Type of cancer | Aggressive care in the last month of life:   - Chemotherapy - Hospital admission - Emergency room visits - ICU admission - Mechanical ventilation | **-Chemotherapy**  OR 0.96 (0.71-1.28) man vs. woman.  **-Hospital admission**  OR 0.89 (0.65-1.23) man vs. woman.  **-Emergency room visits**  OR 0.89 (0.65-1.22) man vs. woman.  **-ICU admission**  OR 0.99 (0.76-1.29) man vs. woman.  **-Mechanical ventilation**  OR 0.82 (0.61-1.11) man vs. woman. | **H** |
| **Ramos-Fernánez et al. 2022**  **Puerto Rico** | Retrospective cohort/10755 | Patients ≥ 18 years diagnosed with primary invasive cancer between 2011-2016 and who died during 2011-2017 | Medicare database | -Age  -Health insurance  -Health region  -Tumor site  -Comorbidity  -Tumor stage at diagnosis | Aggressive care in the last month of life:   - ≥ 2 emergency room visits | **OR 0.89 (0.79-0.99)** woman vs. man. | **H** |
| **Watanabe-Galloway et al. 2014**  **USA** | Transversal/ 34975 | Medicare beneficiaries ≥ 66 years with colorectal cancer who died in 2008 | Medicare database | -Age  -Ethnicity/race  -Socioeconomic satus  -Comorbidity  -Residence: urban/rural | Aggressive care in the last 3 months of life:   - Nº emergency room visits - Nº hospital admissions - Nº ICU admissions | **-Nº emergency room visits**  OR 1.01 (*NS*) woman vs. man.  **-Nº hospital admissions**  OR 1.01 (*NS*) woman vs. man.  **-Nº ICU admissions**  **OR 0.93 (*p*=0.0001)** woman vs. man. | **H** |
| **Palliative care services** | | | | | | | |
| **Adsersen et al. 2021**  **Denmark** | Retrospective cohort/ 5851 | Patients ≥ 18 years diagnosed with pancreatic cancer and who died between 1 July 2011 and 31 December 2018 | Nationa cancer registry and national pallaitive care database | -Age  -Atnicancer treatment  -Metastatic cancer  -Year of death  -Comorbidity  -Region of residence | Acces to specialized palliative care | **OR 0.71** **(0.64-0.79)** man vs. woman | **H** |
| **Barbera et al. 2010**  **Canada** | Retrospective cohort/ 112398 | Patients who died of cancer in Ontario between 2000 and 2004 | Ontario cancer registry, Ontario Health Insurance Plan, Canadian  Institute for Health Information, Ontario Home Care Administrative System and Registered Persons Database | -Age  -Type of cancer  -Comorbidity  -Year of death  -Neighbourhood income  -Rural residence  -Region of residence | Home care:   - Home care in the last 6 months of life - House calls in the last 2 weeks of life | **-Home care in the last 6 months of life**  **OR 1.15 (1.12-1.18)** woman vs. man.  **-House calls in the last 2 weeks of life**  OR 0.97 (0.93-1.00) woman vs. man. | **H** |
| **Burge et al. 2008**  **Canada** | Retrospective cohort/ 7511 | Adults who died of cancer between 1 January 1998-31 December 2003 living within 2 District Health Authorities in the province of Nova Scotia, Canada | Administrative health databases and census | -Age  -Survival  -Education level  -Income  -Ethnic minority  -Long term care resident  -Urban/rural residence  -Comorbidity  -Cancer cause of death  -Inpatient length of stay  -All physician visits  -At least 1 chemo-related visit  -Radiotherapy received last 6 mo of life  -Distance to closest cancer center | Registration with a palliative care program during the last 6 mothns of life | **OR 0.8** **(0.7-0.9)** man vs. woman. | **H** |
| **Craigs et al. 2018**  **UK** | Retrospective cohort/ 2474 | Patients > 18 years who died of cancer between January 2010-February 2012 | Northern and  Yorkshire Cancer Registry, medical records and a clinical information system | Not described | Community and/or hospital palliative care provision | **-Community palliative care**  OR 0.88 (0.68-1.14) man vs. woman.  **-Hospital palliative care**  **OR 0.66 (0.50-0.86)** man vs. woman.  **-Community and hospital palliative care**  **OR 0.66 (0.50-0.87)** man vs. woman. | **H** |
| **Han et al. 2021**  **USA** | Transversal/ 131852 | Patients ≥ 18 years hospitalized with metastatic bladder cancer between 2003 and 2014 | National Inpatient Sample database | -Age  -Race  -Income  -Comorbidity  -Hospital characteristics  -Cancer related factors  -Treatment factors | Inpatient palliative care | OR 1.02 (0.92-1.13) woman vs. man. | **H** |
| **Hegagi et al. 2022**  **USA** | Retrospective cohort/ 6888 | Patients diagnosed with pancreatic cancer between 1 April 2010 and 31 March 2016 | Ontatario cancer registry , home care database and registered person database | -Age  -Rurality  -Income  -Cancer stage  -Comorbidity | Home care (speciallized pallative care units) | OR 1.11 (0.99-1.24) man vs. woman. | **H** |
| **Heller et al. 2019**  **USA** | Retrospective cohort/ 86573 | Patients ≥ 65 with colorectal cancer requiring emergent surgery between 2009-2014 | National Inpatient Sample database | -Age  -Race  -Comorbidity  -Metastasic disease  -Income  -Discharge year  -Hospital bedsize  -Hospital region  -Hospital control  -Teaching hospital  -Urbanicity | Inpatient palliative care consultation | OR 1.0 (0.8-1.1) woman vs. man. | **H** |
| **Jackson et al. 2022**  **USA** | Retrospective cohort/ 23567 | Patients ≥ 18 years hospitalized with esophageal cancer between 2016-2018 | National Inpatient Sample database | -Income  -Age  -Ethnicity/race  -Health insurance  -Hospital characteristics  -Comorbidity  -Length of stay  -Chemotherapy  -Admission type  -Patient disposition | Inpatient palliative care | OR 1.08 (0.98-1.19) woman vs. man. | **H** |
| **Jin et al. 2022**  **USA** | Retrospective cohort/ 48722 | Patients with diagnosis of a malignant primary or secondary central nervous system tumor with at least one neurosurgery encounter between 2003-2011 | Health database (Optum) | -Age  -Race  -Tumor type  -Health insurance  -Surgery post-diagnosis  -Health provider  -Comorbidity | Acces to palliative care and supporte care (home care o social support)   - Inpatient and outpatient palliative care - Home care - Social support | **-Inpatient and outpatient palliative care**  OR 0.981 (*NS*) man vs. woman.  **-Home care**  OR 0.971 (*NS*) man vs. woman.  **-Social support**  **OR 0.850** **(*p*<0.001)** man vs. woman. | **H** |
| **Koroukian et al. 2017**  **USA** | Retrospective cohort/ 835 | Patients ≥ 66 years who died of cancer and received their care exclusively through the traditional fee-for-service system | National databases (Medicare and deaths) and national health survey (Health and Retirement Study) | -Age  -Race  -Marital status  -Income  -Comorbidity  -Tumor site | Hospice care | OR 1.21 (0.86-1.69) woman vs. man. | **H** |
| **Lai et al.**  **2020**  **Taiwan** | Retrospective cohort/ 516409 | Patients >18 years diagnosed with cancer and who died between 1 January 2006 -31 Decembre 2016 | National Health Insurance  Research Database | -Age  -Comorbidity  -Socioeconomic status | Hospice care | **OR 1.22** **(1.21-1.24)** woman vs. man. | **H** |
| **Lee et al. 2021**  **USA** | Retrospective cohort/ 204175 | Patients >18 years with advanced cancer hospitalized between 2012-2014 | National Inpatient Sample database | -Age  -Race  -Socioeconomic status  -Comorbidity  -Surgical procedure  -Chemotherapy or radiotherpay  -Gastrostomy tube  -Parenteral nutrition  -Hemodialysis  -Posoperative complications  -Length of stay  -Hospital characteristics | Inpatient palliative care consultation | **OR 1.03** **(1.002-1.061)** woman vs. man. | **H** |
| **Lindskog et al. 2022**  **Sweden** | Retrospective cohort/ 780 | Patients >18 years diagnosed with malignant brain neoplasm who died between 2015-2019 | Stockholm regional database | -Age  -Socioeconomic status  -Comorbidity  -Nursing home resident | Access to specialized palliative care during the last 3 months of life | OR 1.02 (0.69-1.52) woman vs. man. | **H** |
| **Maddison et al. 2012**  **Canada** | Retrospective cohort/ 1201 | Patients ≥ 20 years who died of colorectal cancer between 1 January 2001 and 31 March 2008 | National cancer registry and health and palliative care databases | -Residence: urban/rural  -Age  -Income  -Travel distance  -Comorbidity  -Tumor stage  -Long-term care residency  -Emergency room visits in the last month of life  -Place of death  -Year of death | Access to a palliative care program:   - Registration in a palliative care program - Registration ≥60 days prior to death | **-Registration in a palliative care program**  OR 0.85 (0.5-1.3) woman vs. man.  **-Registration ≥60 days prior to death**  OR 1.52 (1.0-1.24) woman vs. man. | **H** |
| **Nayar et al. 2014**  **USA** | Transversal/ 91039 | Medicare beneficiaries ≥ 66 years with lung cancer who died in 2008 | Medicare database | Not described | Access to hospice care:   - Hospice care use - Hospice enrollment in last 3 days | **-Hospice care use**  **OR 1.22 (*p*=0.0001)** woman vs. man.  **-Hospice enrollment in last 3 days**  **OR 0.88** **(*p*=0.0001)** woman vs. man. | **M** |
| **Okafor et al. 2017**  **USA** | Transversal/ 513440 | Patients hospitalized with metastatic gastrointestinal cancer in 2012 | National Inpatient Sample database | -Age  -Race  -Health insurance  -Income  -Hospital region  -Hospital bedsize  -Hospital teaching status  -Hospital ownership | Inpatient palliative care | **OR 1.12 (*p=*0.002)** woman vs. man. | **H** |
| **Rubens et al. 2019**  **USA** | Retrospective cohort/ 4732172 | Patients ≥18 years hospitalized with advanced cancer between 2005-2014 | National Inpatient Sample database | -Age  -Race  -Socioeconomic status  -Cancer type  -Health insurance  -Hospital region  -Hospital bedsize  -Hospital teaching status  -Do not resuscitate status  -Previous radiotherapy  -Chemotherapy, mechanical ventilation, parenteral o enteral nutrition, hemodialysis | Inpatient palliative care | OR 1.04 (0.98-1.06) woman vs. man. | **H** |
| **Sharma et al. 2015**  **USA** | Retrospective cohort/ 6288 | Patients with metastatic cancer admitted to Northwerstern Memorial Hospital, Chicago, between 1 January 2009 -31 December 2010 | Medical records | -Health insurance  -Severity of illness  -Marital status  -Prior hospitalization  -Ethnicity/race | Inpatient palliative care consultation | OR 1.00 (0.86-1.15) woman vs. man. | **M** |
| **Sharp et al. 2018**  **Sweden** | Transversal/ 1872 | Patients ≥18 years diagnosed between 2014-2016 with primary gyneacological, hematological, head and neck and upper gastrointestinal cancer, living within the Stockholm Gotland area | Questionnaire | -Sex  -Cancer type  -Place of birth  -Occupation status  -Education level  -Living situation  -Treatment and type of treatment | Acceso to supportive care:   - Contact nurse - Individual written plans - Patient advocacy groups - Referred to palliative care | **-Contact nurse**  OR 1.11 (0.84-1.46) man vs. woman.  **-Individual written plans**  **OR 1.33** **(1.01-1.73)** man vs. woman.  **-Patient advocacy groups**  OR 1.01 (0.76-1.36) man vs. woman.  **-Referred to palliative care**  OR 1.05 (0.77-1.45) man vs. woman. | **H** |
| **Watanabe-Galloway et al. 2014**  **USA** | Transversal/ 34975 | Medicare beneficiaries ≥ 66 years with colorectal cancer who died in 2008 | Medicare database | -Age  -Ethnicity/race  -Socioeconomic satus  -Comorbidity  -Residence: urban/rural | Access to hospice care:   - Hospice care use - Hospice enrollment in last 3 days | **- Hospice care use**  **OR 1.12 (*p*=0.0001)** woman vs. man.  **-Hospice enrollment in last 3 days**  **OR 0.91 (*p*=0.0001)** woman vs. man. | **H** |
| **Advance care planning** | | | | | | | |
| **Hu et al. 2021**  **China** | Transversal/ 258 | Patients diagnosed with lung cancer hospitalized between October 2017 and November 2018 | Questionnaire and medical records | -Age  -Marital status  -Tumor type  -Tumor stage  -Treatment status  -Number of children  -Economic burden  -Quality of Life scale scores | Palliative care preferences:   - Initiating end of lfe decisions by others (health care profesionals) | **OR 2.743** **(2.243-3.285)** woman vs. man. | **H** |
| **Koffman et al. 2007**  **UK** | Transversal/ 252 | Oncology patients who were receiving curative treatments at two district general hospitals in north-west London between December 2004 and April 2005 | Survey and national register | -Age | Palliative care knowledge**:**   - Recognises term palliative care - Understands the role of Macmillan nurses | **-Recognises term palliative care**  OR 1.7 (0.71-4.23) woman vs.man.  **-Understands the role of Macmillan nurses**  OR 2.0 (0.95-3.94) woman vs. man. | **M** |
| **O’Mahony et al. 2021**  **USA** | Transversal/ 308 | Patients aged ≥ 55 years with a cancer diagnosis who were being seen in a palliative care ambulatory clinic | Questionnaire and medical records | -Age  -Race/ethnicity  -Education level  -Income  -Marital status  -Religious affiliation  -Spirituality  -Terminal illness awareness | Palliative care preferences:   - Treatment preferences - Cardiopulmonary resuscitation preferences - Mechanical ventilation preferences | **-Treatment preferences**  Regression coefficient -0.221 (*NS*) man vs. woman.  **-Cardiopulmonary resuscitation preferences**  Regression coefficient -0.363 (*NS*) man vs. woman.  **-Mechanical ventilation preferences**  **Regression coefficient** -**0.620 (*p*<0.05)** man vs. woman. | **M** |
| **Saeed et al. 2018**  **USA** | Transversal/ 383 | Patients ≥ 21 years with advanced cancer (stage IV cancer non-hematologic cancer or stage III cancer) | Survey | -Age  -Race  -Health insurance  -Perceibed financial strain  -Cancer type  -Marital status  -Living situation  -Quality of life (MQOL)  -Distress  -PEACE scale  -Physical and social well-being scale (FACT-G)  -Presence or absence of caregiver  -Study site | Preference for palliative care | **OR 2.69** **(1.51-4.79)** woman vs. man. | **M** |
| **Saeed et al.**  **2019**  **USA** | Transversal/ 265 | Patients ≥ 21 years with advanced cancer (stage IV cancer non-hematologic cancer or stage III cancer) | Survey | -Age  -Race  -Perceibed financial strain  -Income  -Education level  -Doctor’s subspecialty  -Study site | Completion of advance directives | Regression coefficient -0.01 (-0.20-0.17) man vs. woman. | **M** |
| **Place of death** | | | | | | | |
| **Barbera et al. 2010**  **Canada** | Retrospective cohort/ 112398 | Patients who died of cancer in Ontario between 2000 and 2004 | Ontario cancer registry, Ontario Health Insurance Plan, Canadian  Institute for Health Information, Ontario Home Care Administrative System and Registered Persons Database | -Age  -Type of cancer  -Comorbidity  -Year of death  -Neighbourhood income  -Rural residence  -Region of residence | Acute care death | **OR 0.88 (0.85-0.91)** woman vs. man. | **H** |
| **Burge et al.**  **2005**  **Canada** | Retrospective cohort/ 13652 | Adult patients residing in Nova Scotia and who died of cancer between 1992-1997 | Administrative health databases and census | -Age  -Cancer type  -Income  -Year of death  -Survival  -Received a visit in long term-care  -Lengh of stay | Out of hospital death | **OR 1.24 (1.12-1.37)** woman vs. man. | **H** |
| **D’Angelo et al. 2020**  **Italy** | Retrospective cohort/ 13656 | Patients > 18 years residents in Lazio region who died of advanced cancer between January 2012-December 2016 | Hospital discharge register and electronic medical records | -Age  -Ethnicity  -Education level  -Citizenship  -Area of residence  -Distance to closest specialised palliative service  -Cancer type  -Survival | Home or hospice care facility death vs. hospital death | OR 0.92 (0.81-1.03) man vs. woman. | **H** |
| **Gatrell et al. 2003**  **Inglaterra** | Transversal/ 6900 | Patients who died of cancer between 1993-2000 | National registers | Not described | - Home - Acute care hospital - Hospice - Nursing home | **-Home**  **OR 0.812 (0.717-0.919)** woman vs. man.  **-Acute care hospital**  **OR 0.876** **(0.786-0.976)** woman vs. man.  **-Nursing home**  **OR 1.391 (1.150-1.683)** woman vs. man. | **M** |
| **Hegagi et al. 2022**  **USA** | Retrospective cohort/ 6888 | Patients diagnosed with pancreatic cancer between 1 Abpril 2010- 31 March 2016 | National cancer registry and home care database | -Age  -Rurality  -Income  -Cancer stage  -Comorbidity  -Home care category | Out of hospital death | OR 1.00 (0.88-1.14) man vs. woman. | **H** |
| **Hunt et al.**  **2001**  **Australia** | Transversal/ 29230 | Patients who died of cancer in South Australia between 1990-1999 | State cancer registry | -Age  -Race  -Country of birth  ­-Place of residence  -Socieconomic status  -Survival  -Year of death  -Cancer type | - Metropolitan private hospital - Country hospital - Hospice - Nursing home - Home - Metropolitan public hospital | **Place of death vs. metropolitan public hospital**  **-Metropolitan private hospital**  **OR 1.35** (1.23-1.49) woman vs. man.  **-Country hospital**  **OR 1.23** (1.10-1.34) woman vs. man.  **-Hospice**  **OR 1.24** (1.14-1.38) woman vs. man.  -**Nursing home**  **OR 1.79** (1.61-1.99) woman vs. man.  **-Home**  OR 1.05 (0.96-1.15) woman vs. man. | **H** |
| **Koroukian et al. 2017**  **USA** | Retrospective cohort/ 835 | Patients ≥ 66 years who died of cancer and received their care exclusively through the traditional fee-for-service system | National databases (Medicare and deaths) and national health survey (Health and Retirement Study) | -Age  -Race  -Marital status  -Income  -Comorbidity  -Tumor site | In hospital death | OR 0.81 (0.54-1.20) woman vs. man. | **H** |
| **Li et al. 2020**  **China** | Transversal/ 894 | Residents who died from cancer from July 2015 to June 2017 in Yichang, China | Yichang health database | -Age  -Education level  -Health insurance  -Marital status | In hospital death | OR 1.16 (0.80-1.67) woman vs. man. | **H** |
| **Lindskog et al. 2022**  **Sweden** | Retrospective cohort/ 780 | Patients >18 years diagnosed with malignant brain neoplasm who died between 2015-2019 | Stockholm regional database | -Age  -Socioeconomic status  -Comorbidity  -Nursing home resident | Acute care hospital death | OR 0.56 (0.28-1.12) woman vs. man. | **H** |
| **López-Valcarcel et al. 2019**  **Spain** | Transversal/ 79506 | Adult patients ≥24 years who died of cancer in Spain during 2015 | National death register, economic database and palliative care database | Not described | - Home vs. hospital - Long term care centre vs. home or hospital - Hospital vs. home or long term care centre | **-Home**  **Regression coefficient 0.033 (*p*<0.01)** woman vs. man.  **-Long term care centre**  **Regression coefficient 0.0090 (*p*<0.01)** woman vs. man.  **-Hospital**  **Regression coefficient -0.0353 (*p*<0.01)** woman vs. man. | **H** |
| **Maddison et al. 2012**  **Canada** | Retrospective cohort/ 1201 | Patients ≥ 20 years who died of colorectal cancer between 1 January 2001 and 31 March 2008 | National cancer registry and health and palliative care databases | -Residence: urban/rural  -Age  -Income  -Travel distance  -Comorbidity  -Tumor stage  -Long-term care residency  -Emergency room visits in the last month of life  -Place of death  -Year of death | In hospital death | OR 1.19 (0.9-1.6) woman vs. man. | **H** |
| **Neergaard et al. 2012**  **Denmark** | Transversal/ 599 | Adult patients living in Aarhus (Denmark) who died of cancer between 1 March and 30 November 2006 | National registers | -Age  -Diagnosis  -Marital status  -Children residing at home  -Housing space  -Inmigrant  -Conctact with general practitioner  -Lengh of hospital stay  -Specialist team involvement | Home death | PR 1.15 (0.97-1.36) man vs. woman. | **H** |
| **Öhlén et al.**  **2017**  **Sweden** | Transversal/ 20710 | Patients who died of cancer in 2012, Sweden | National registers | -Age  -Cancer type  -Marital status  -Educational level  -Urbanicity  -Health care region | - Home - Hospital - Nursing home | **-Hospital vs. home (place of residence)**  OR 0.99 (0.91-1.08) woman vs. man.  **-Nursing home vs. home (place of residence)**  OR 1.09 (0.98-1.21) woman vs. man.  **-Hospital vs. nursing home (place of residence)**  OR 0.86 (0.52-1.42) woman vs. man. | **H** |
| **Papke et al. 2007**  **Germany** | Transversal/ 2316 | Patients who died of cancer between 1997-2003 | Health Office of the administrative district “*Sächische Schweiz*” | Not described | Nursing home | **OR 0.48 (0.32-0.73)** man vs. woman. | **M** |
| **Sedhom et al. 2021**  **USA** | Retrospective cohort/ 3182707 | Patients >65 years who died of lung, colorectal, prostate, pancreatic and breast cancer between 2003 -2017 | National register | -Age  -Year of death  -Ethnicity/race  -Primary cancer diagnosis | - Hospice facility - Hospital - Nursing home - Home | **Place of death vs. home**  **-Hospice facility**  **OR 0.94 (0.93-0.95)** man vs. woman.  **-Hospital**  **OR 1.12 (1.11-1.13)** man vs. woman.  **-Nursing home**  **OR 0.83 (0.83-0.84)** man vs. woman. | **H** |

NS: Not signifiant; USA: Unit States of America; ICU: Intensive Care Unit; OR: *odds ratio*; RR: relative risk; CI: confidence interval

*Statistically significant results highlighted in bold (p<0.05).

§Results of the evaluation of methodological quality using the NewCastle-Ottawa Scale (NOS).Categorized in high (H), moderate (M) and low (L).
